# Humanized Patient-derived Xenograft Models of Disseminated Ovarian Cancer Recapitulate Key Aspects of the Tumor Immune Environment within the Peritoneal Cavity

**DOI:** 10.1158/2767-9764.CRC-22-0300

**Published:** 2023-02-22

**Authors:** Mara P. Steinkamp, Irina Lagutina, Kathryn J. Brayer, Fred Schultz, Danielle Burke, Vernon S. Pankratz, Sarah F. Adams, Laurie G. Hudson, Scott A. Ness, Angela Wandinger-Ness

**Affiliations:** 1 Department of Pathology, University of New Mexico School of Medicine, Albuquerque, New Mexico.; 2 Comprehensive Cancer Center, University of New Mexico, Albuquerque, New Mexico; 3 Analytical and Translational Genomics Shared Resource, Comprehensive Cancer Center, University of New Mexico, Albuquerque, New Mexico.; 4 Department of Internal Medicine, University of New Mexico School of Medicine, Albuquerque, New Mexico.; 5 Biostatistics Shared Resource, Comprehensive Cancer Center, University of New Mexico, Albuquerque, New Mexico.; 6 Department of Obstetrics and Gynecology, University of New Mexico School of Medicine, Albuquerque, New Mexico.; 7 Department of Pharmaceutical Sciences, University of New Mexico School of Medicine, Albuquerque, New Mexico.; 8 Department of Molecular Genetics and Microbiology, University of New Mexico School of Medicine, Albuquerque, New Mexico.

## Abstract

The importance of the immune microenvironment in ovarian cancer progression, metastasis, and response to therapies has become increasingly clear, especially with the new emphasis on immunotherapies. To leverage the power of patient-derived xenograft (PDX) models within a humanized immune microenvironment, three ovarian cancer PDXs were grown in humanized NBSGW (huNBSGW) mice engrafted with human CD34^+^ cord blood–derived hematopoietic stem cells. Analysis of cytokine levels in the ascites fluid and identification of infiltrating immune cells in the tumors demonstrated that these humanized PDX (huPDX) established an immune tumor microenvironment similar to what has been reported for patients with ovarian cancer. The lack of human myeloid cell differentiation has been a major setback for humanized mouse models, but our analysis shows that PDX engraftment increases the human myeloid population in the peripheral blood. Analysis of cytokines within the ascites fluid of huPDX revealed high levels of human M-CSF, a key myeloid differentiation factor as well as other elevated cytokines that have previously been identified in ovarian cancer patient ascites fluid including those involved in immune cell differentiation and recruitment. Human tumor-associated macrophages and tumor-infiltrating lymphocytes were detected within the tumors of humanized mice, demonstrating immune cell recruitment to tumors. Comparison of the three huPDX revealed certain differences in cytokine signatures and in the extent of immune cell recruitment. Our studies show that huNBSGW PDX models reconstitute important aspects of the ovarian cancer immune tumor microenvironment, which may recommend these models for preclinical therapeutic trials.

**Significance:**

huPDX models are ideal preclinical models for testing novel therapies. They reflect the genetic heterogeneity of the patient population, enhance human myeloid differentiation, and recruit immune cells to the tumor microenvironment.

## Introduction

The search for novel treatment regimens for ovarian cancer would benefit from preclinical models that represent the genetic heterogeneity of the patient population, while recapitulating the unique ovarian cancer tumor microenvironment including the tumor-promoting immune cell populations. Previous studies have shown that patient-derived xenograft (PDX) models retain the genetic heterogeneity of the primary tumor and recapitulate the original tumor morphology (1–4). Use of multiple PDX models is advantageous for sampling the heterogeneity across the patient population and increases the translational potential of a given intervention (4–6). However, PDX models growing in immunocompromised mice lack the immune components of the tumor microenvironment.

Irradiated, immunocompromised NSG mice engrafted with CD34^+^ hema-topoietic stem cells (HSC) yield primarily human B and T cells and are the standard humanized mouse model for oncology studies. However, this model has a low representation of myeloid populations, particularly macrophages (7). The lack of myeloid cells is a critical shortcoming in the standard humanized NSG model particularly for ovarian cancer research. Ovarian cancer cells produce cytokines that recruit macrophages to tumors and skew macrophage polarization toward an anti-inflammatory state (8). In turn, tumor-associated macrophages (TAM) release a range of factors such as EGF that stimulate cancer cell proliferation and metastasis (9). TAMs make up 30%–50% of the cells found in malignant ascites (10). Therefore, it is critical that TAMs be present in humanized ovarian cancer PDX models.

With the recognition that myeloid cells are critical in tumor biology and immunity, attempts have been made to enrich myeloid cells in humanized models. New strategies make use of transgenic mice that constitutively express human cytokines to improve myeloid differentiation. The NSG-SGM3 transgenic model that constitutively expresses human GMCSF, has a larger number of myeloid progenitor cells in the bone marrow, but does not show an increase in differentiated myeloid populations in the peripheral blood (11). The MISTRG mouse model that constitutively expresses human M-CSF has higher numbers of myeloid cells in the blood, but rapidly develops anemia, which can reduce the window for performing cancer studies (12, 13). Thus, these strategies are not yet ideal.

Recent studies examining the anticancer effects of human immune cells in ovarian cancer xenograft models have adoptively transferred subsets of human immune cells including allogeneic natural killer (NK) cells (14), allogeneic tumor-primed human T cells (15), and autologous mature T cells from patient tumors (16). These models do not reconstitute the full complement of human immune cells within the tumor microenvironment. One study engrafted autologous peripheral blood mononuclear cells (PBMC) or tumor-associated leukocytes (TAL) into a subcutaneous or intraperitoneal ovarian cancer PDX model (17). However, this model requires the isolation of autologous immune cells, which is not always possible. Thus, this model does not translate easily to large-scale therapeutic studies. Importantly, the autologous cells (TALs or PBMCs) contain human T cells that have matured in the patient and are therefore more likely to cause GvHD compared with our CD34^+^ HSC-engrafted humanized mice where GvHD manifests at much later timepoints (>24 weeks after engraftment; ref. 18). Our aim was to develop a disseminated ovarian cancer PDX model with a more complete humanized immune system (HIS) to better represent the unique immune microenvironment within the peritoneal cavity.

Here, we present a strategy for ovarian cancer growth in humanized mice that results in myeloid engraftment, differentiation, and tumor infiltration. We establish disseminated ovarian cancer PDX models in humanized NBSGW (huNBSGW) mice engrafted with human cord blood–derived CD34^+^ HSCs. The NBSGW (NOD.Cg-KitW-41J Tyr + Prkdcscid Il2rgtm1Wjl/ThomJ) strain on the NSG background carries a *c-kit* mutation that weakens the ability of mouse HSCs to compete with engrafted human cells. huNBSGW demonstrate efficient humanization and increased CD33^+^ myeloid progenitor cells in the bone marrow and CD11b^+^ myeloid cells and CD1a^+^ dendritic cells within the spleen without the need for pre-engraftment irradiation (19, 20). By engrafting ovarian cancer PDX models into huNBSGW mice, we can take advantage of the genetic heterogeneity of the PDX models, while restoring the complex interactions between human cancer cells and human immune cells. Cytokine profiling and evaluation of tumor-infiltrating immune cells in multiple human PDX (huPDX) models demonstrated a reconstitution of the peritoneal tumor immune environment in these models.

## Materials and Methods

### Banking Patient Ovarian Cancer Spheroids and Establishing PDX Models From Malignant Ascites

Malignant ascites from patients with ovarian cancer were collected during cytoreductive surgery at the University of New Mexico Comprehensive Cancer Center (UNMCCC, Albuquerque, NM). Acquisition of patient samples was approved by the UNM Health Science Center Institutional Review Board (protocol no. INST1509). Studies were conducted in accordance with the U.S. Common Rule. Written consent was obtained from all patients from which ascites was collected. Ascites samples were centrifuged and cell-free ascites fluid was stored at −80°C for cytokine analysis. After red blood cell lysis using Ammonium Chloride Solution (STEMCELL Technologies), cancer spheroids were isolated and 20 × 10^6^ ovarian cancer cells were injected into the peritoneal cavity of NSG mice (RRID: IMSR_JAX:005557) to establish orthotopic PDX models of disseminated ovarian cancer. Isolated cancer cells transferred directly into mice were not tested for *Mycoplasma*. However, our lab regularly tests cell lines for *Mycoplasma* using the MycoAlert PLUS detection kit (Lonza) and have not detected any *Mycoplasma* contamination in our cultures. Mice were euthanized at a humane endpoint when mice exhibited abdominal distention from ascites accumulation or showed signs of wasting. Solid tumors and ascites fluid were collected. PDX samples were cryopreserved in 95% FBS/5% DMSO or injected into new NSG mice for passaging. All mouse procedures were approved by the UNM Animal Care and Use Committee (protocol no. 18-200722-HSC), in accordance with NIH guidelines for the Care and Use of Experimental Animals. PDX lines were authenticated by short tandem repeat (STR) profile analysis (ATCC) in which the PDX profile was compared with the primary patient ascites sample. For all three PDX lines used in this study (PDX3, 9, and 18), all PDX alleles are a match for patient alleles indicating that the PDX were derived from the patient samples. PDX3 was missing two alleles that were present in Patient 3, which could be due to clonal selection in the development of the PDX line. During PDX development, lines were monitored for lymphoproliferative lesions of both human and mouse origin that can contaminate PDX lines. PDX tumor sections were stained for a human mitochondrial marker to ensure PDX lines were of human origin. At necropsy, PDX-engrafted mice were examined for splenomegaly, an indication of lymphoproliferative lesions. PDX ascites fluid was also examined for the presence of characteristic ovarian cancer multicellular aggregates (spheroids) with no evidence of contaminating small single cells that would indicate lymphomas. All three PDX that are included in this study had solid tumors that stained positive for the human mitochondrial marker, had STR profiles that matched the original patient ascites sample, and presented at end stage with malignant ascites that contained cancer spheroids. Multidimensional scaling plots of RNA sequencing (RNA-seq) data from primary ascites sample and paired PDX samples of four PDX lines including PDX3 and PDX9, showed similarity within samples that have the same patient origin suggesting that PDX retain the global expression pattern of the original patient samples (Supplementary Fig. S1A). Previous studies have suggested that low PDX passages retain the morphologic and genetic characteristics of the original patient sample (21, 22). Prior work has examined the genetic stability of ovarian cancer PDX and has found that PDX retain morphologic features as well as copy-number variations of the patient tumor. They also retain multiple clones from the original patient sample rather than demonstrating a selection of one clone during PDX formation (3).

### RNA Isolation and RNA-seq of Ovarian Cancer Samples

RNA-seq analysis was performed on primary ovarian cancer solid tumor, primary ascites cells, non-huPDX solid tumor, and non-huPDX ascites cells for select patients. Formalin-fixed paraffin-embedded (FFPE) sections of matched patient solid tumor were obtained through the UNM Human Tissue Repository. For patient solid tumor samples, RNA isolation was performed by the UNMCCC Analytical and Translational Genomics (ATG) Shared Resource, as described previously (23, 24). Briefly, total RNA was isolated from slide-mounted FFPE sections using the RNeasy FFPE kit (Qiagen). For PDX samples, snap-frozen solid tumor was incubated in RNAlater-ICE Frozen Tissue Transition Solution (Invitrogen) for 24 hours at −20°C prior to DNA/RNA extraction with AllPrep DNA/RNA Mini Kit (Qiagen). Cryopreserved patient ascites cells and PDX cancer spheroids were thawed and rinsed in serum-free RPMI before RNA/DNA extraction. cDNA synthesis and library preparation were performed in the UNMCCC ATG Shared Resource using the SMARTer Universal Low Input RNA Kit for Sequencing (Clontech) and the Ion Plus Fragment Library Kit (Life Technologies) as described previously (24).

### RNA-seq Analysis

Low-quality and non-human RNA-seq reads were identified and removed from the analysis pipeline using the Kraken suite of quality control tools (25, 26). High-quality, trimmed, human RNA-seq reads were aligned to the human genome (GRCh37; hg19) using TMAP (v5.0.7) and gene counts were calculated using High-throughput sequence analysis in Python (HT-Seq) as described previously (24). Gene set enrichment analysis (GSEA, RRID:SCR_005724) comparing PDX samples, primary ovarian cancer ascites, and primary ovarian cancer solid tumors was analyzed using the GSEA software (http://www.gsea-msigdb.org/gsea/index.jsp; ref. 27). A total of 1,742 genes in the ovarian cancer dataset were compared with 7,871 gene sets from the Molecular Signature Database after filtering out for gene set size (minimum 15, maximum 500 genes/set). For comparison of PDX and primary ovarian cancer gene expression, nine solid tumor or ascites PDX samples were compared with 13 primary or omental ovarian tumors, and 10 primary ascites samples. Samples were divided into three groups: PDX, primary ascites, or primary solid tumor. Genes with significantly altered expression between groups were tabulated.

Deconvolution of normalized gene expression data was performed using the publicly available Carcinoma Ecotyper software (https://ecotyper.stanford.edu/carcinoma; ref. 28). Luca and colleagues analyzed 16 select tumor types, including ovarian serous cystadenocarcinoma, to identify the cellular composition and cell states based on gene expression clusters identified from single-cell RNA-seq datasets. The gene expression clusters can then be used to perform deconvolution on bulk RNA-seq tumor samples. Our ovarian cancer expression data from four PDX ascites samples, five PDX tumors, eight patient ascites samples, and 14 patient tumors were analyzed using this program. Output is given as the abundance by state for 12 cell types including immune cell types, cancer epithelial cells, endothelial cells, and fibroblasts. The average estimated abundance of monocytes/macrophages, CD4^+^ T cells, CD8^+^ T cells, and cancer epithelial cells by state is presented for the four sample types.

### huNBSGW Mice

Cryopreserved human cord blood–derived CD34^+^ cells (purity >90% as determined by flow cytometry, and HIV-1 and 2, hepatitis B and C negative, STEMCELL Technologies) were rapidly thawed at 37°C, resuspended in media (RPMI +1% human serum albumin), centrifuged (300 × *g*, 10 minutes at room temperature), and rinsed in media. After centrifugation, the cell pellet was resuspended in 1 mL of Stemline II Hematopoietic Stem Cell Expansion Medium (Sigma-Aldrich), supplemented with 0.1 μg/mL Human Recombinant SCF (STEMCELL Technologies). Cells were incubated overnight in a standard CO_2_ incubator at 5% CO_2_. Prior to engraftment, cells were centrifuged (300 × *g* for 10 minutes at room temperature) and resuspended in PBS. 2.5 × 10^5^ CD34^+^ cells were administered by retro-orbital injection into 3–4 weeks old female NBSGW mice (RRID:IMSR_JAX:026622). Pooled donor samples were used to limit between-donor variability. Therefore, we did not HLA-match the donor HSCs to the PDXs. Studies with human xenografts in CD34^+^ HSC-engrafted humanized NSGs have shown that partially matched donor cells allow growth of human tumors (7). Peripheral blood was drawn from huNBSGW mice at 8 weeks after engraftment to characterize the percentage of human immune cells [% human CD45^+^ cells/(total mouse CD45^+^ cells + human CD45^+^ cells)] present in the peripheral blood to ensure optimal humanization of 25% or greater as in Wang and colleagues (7). Human immune cell subpopulations were also assessed by flow cytometry (Supplementary Fig. S2A). Antibodies are listed in Supplementary Table S1. Mice with greater than 25% human CD45^+^ cells in peripheral blood were considered optimally humanized. Percent humanization at 8 weeks after engraftment ranged from 40% to 84% (Supplementary Fig. S2B). Mice were divided into three groups for engraftment of three PDX models. Humanization was not significantly different between the groups.

### huPDX Mice

Three huNBSGW mice per PDX were injected 10 weeks after CD34^+^ cell engraftment with fresh ovarian cancer spheroids from three PDX models [PDX3 passage 2 (P2), PDX9 P4, and PDX18 P6]. Three age-matched NBSGW control mice per PDX were injected as non-humanized controls. A total of 5 × 10^6^ PDX cells/mouse were injected intraperitoneally to seed orthotopic disseminated ovarian cancer. Mice were weighed weekly and monitored for wasting or abdominal distention. Mice were also observed for signs of anemia that can develop in humanized mice with improved human myeloid cell engraftment. Once mice showed signs of disease, they were monitored daily and sacrificed at a humane endpoint. Peripheral blood was collected at 2 and 6 weeks after tumor engraftment and blood and ascites fluid were collected at endpoint. All tumors were removed and the mass of the total tumor burden was recorded. One huPDX9 mouse and one non-huPDX18 mouse died overnight and samples could not be collected.

### IHC of Humanized Solid Tumors

Solid tumors and spleens from PDX models (*n* = 2–3 per group) were formalin fixed at necropsy. huPDX spleens were used for IHC optimization of anti-human antibodies in mouse tissue (Supplementary Fig. S5C). Fixed tissue was paraffin embedded and sectioned by the UNM HTR and IHC was performed on the Ventana Discovery Ultra Platform using immunoperoxidase labeling. Serial sections were processed for each marker. Antibodies are listed in Supplementary Table S1. For digital pathology and HALO analysis (Indica Labs), quantitative analysis algorithms were optimized to detect intratumoral infiltrating human immune cells. Immune cells were classified as intratumoral or extratumoral with a tumor tissue mask based on labeling with a human mitochondrial marker or hematoxylin and eosin (H&E) staining. After omitting areas with obvious staining artifacts, all cells within the tumor were identified on the basis of the nuclear hematoxylin counterstain and positive cells were tallied as weak, medium, or strong staining. One huPDX3 sample contained no tumor tissue and was not included in the analysis. The average number of positive cells/100,000 cells were plotted for each group. For analysis of tumor vascularization, tumor vessels were labeled with anti-mouse CD31 antibody and slides were digitally scanned. The vessel area was determined using the tissue classifier analysis software on the HALO platform. Vascular density was reported as percent vascular area and was calculated on the basis of analysis of whole sections from huPDX and non-huPDX tumors.

### Cytokine Analysis of Ascites Fluid and Peripheral Blood Plasma

For cytokine array analysis, frozen cell-free ascites fluid and peripheral blood plasma samples were submitted to Eve Technologies for analysis on their Human Cytokine/Chemokine Array 48-Plex. Forty-eight cytokines, chemokines, and growth factors were analyzed in duplicate from 100 μL of sample. Human M-CSF and VEGF-A levels were measured in patient and PDX cell-free ascites fluid using the Human M-CSF ELISA Kit or Human VEGF-A ELISA kit, respectively (RayBiotech). huPDX cytokine levels were compared with published LINCOplex microarray data from paired ascites and plasma samples from ovarian cancer (29). Macrophage-derived chemokine (MDC) values were compared with published data from 93 patients with ovarian cancer (30). For uniformity, average and SEM for MDC levels were estimated from the listed median and interquartile range using an online estimator (31).

### Statistical Analyses

Statistical methods were applied to the cytokine array data to determine whether observed cytokine measurements differed according to two primary factors: ascites versus plasma sample source and huPDX versus non-huPDX. First, a linear mixed-effects model was fit to the data, treating specific cytokines as a repeated measure factor. This made it possible to test the global significance of cytokine-specific differences among the various grouping factors. It also enabled the estimate of average between-group differences across all cytokines. After identifying factors where there was evidence of statistical differences between groups, we fit a separate analysis of variance model to each cytokine to test the significance of the between-group differences and estimate the within-group means for each cytokine. For survival curve comparisons, analyses were performed using GraphPad Prism software (RRID:SCR_002798) and used a log-rank Mantel–Cox test to determine significant differences between huPDX and non-huPDX. Statistical analysis for differences in tumor burden and ascites volume between huPDX and non-huPDX and for differences in cell states for deconvolution analysis was assessed using unpaired Students *t* tests.

### Data Availability

The data generated in this study are available within the article and its Supplementary Data.

## Results

### Ovarian Cancer PDX in Immunocompromised Mice Lack Immune Cells That are Crucial for Establishing a Supportive Tumor Microenvironment in Solid Ovarian Tumors and Ascites Fluid

To establish ovarian cancer PDX models that sample the genetic heterogeneity of the UNMCCC patient population, cancer spheroids isolated from the malignant ascites of patients with confirmed high-grade serous ovarian cancer (HGSOC) were engrafted into the peritoneal cavity of NSG mice. The purpose of the current study was to examine the impact of a humanized immune system on the disease progression and cytokine milieu of PDX models developed from disseminated HGSOC. Our PDX models were specifically developed from the ascites fluid of patients with HGSOC with an adequate number of cancer cells for PDX engraftment and selected on the basis of their unique genetic backgrounds and mutational burden, as well as their robust growth in mice. All patient samples collected were stage 3b, 3c, or 4, which is reflective of the fact that HGSOC is the most common and the most aggressive form of ovarian cancer. PDX developed from 6 of 9 patient samples for a 67% take rate, similar to rates reported in the literature for solid ovarian cancer PDX (68%–74%) and higher than reported rates for spheroid-derived PDX orthotopic tumors (31%; refs. 3, 4, 6, 32). Our PDX models developed tumors within the omentum and mesentery, near the ovaries, and in the perigonadal fat pads with occasional spread to the liver and diaphragm. The dissemination throughout the peritoneal cavity is similar to that seen in patients with ovarian cancer (33, 34).

**FIGURE 1:**
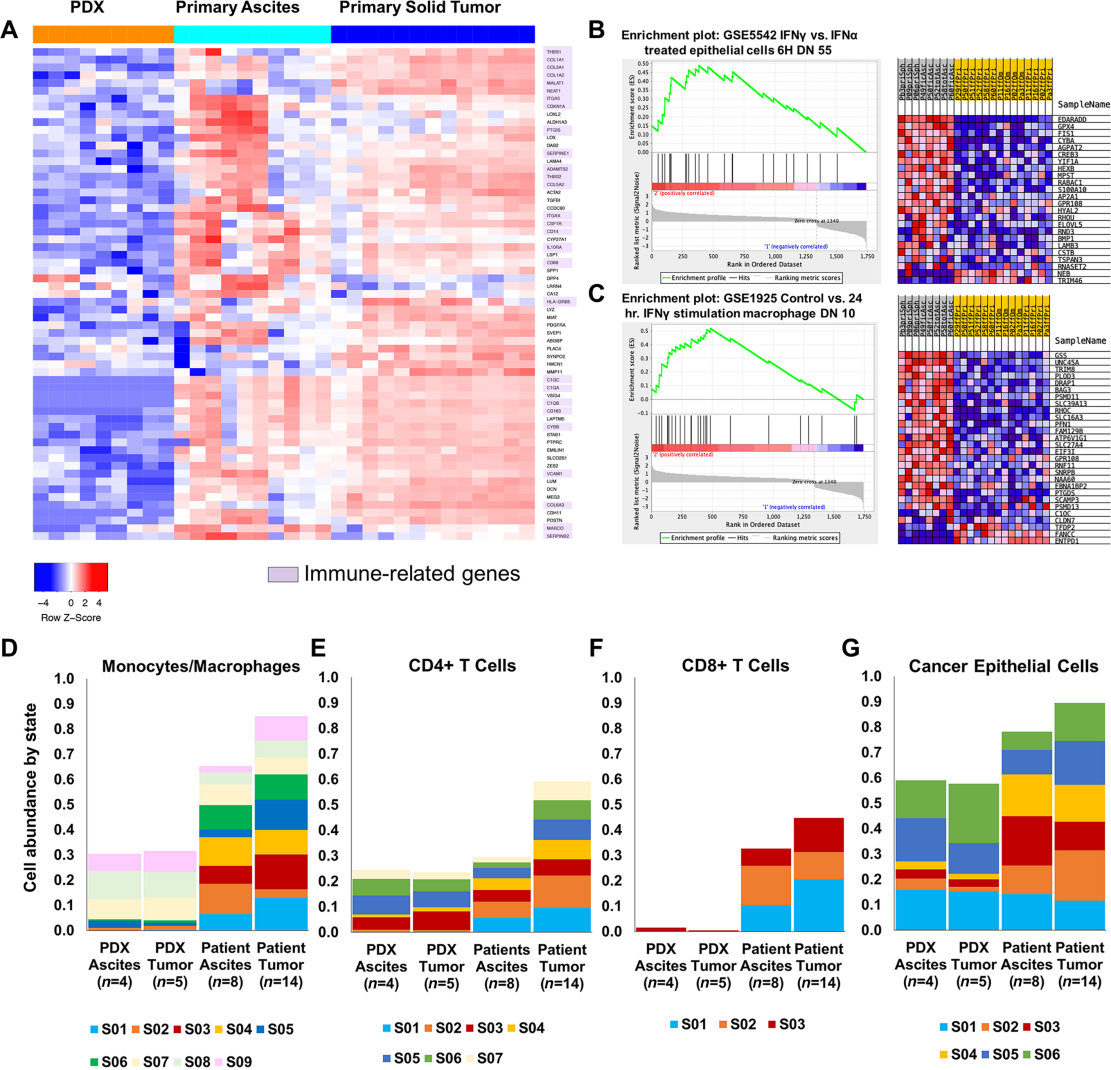
Analysis of RNA-seq data comparing ovarian cancer patient samples to non-huPDX samples highlights the importance of immune cells in the patient samples. **A,** Of the differentially expressed genes that show significantly lower expression in non-huPDX samples (orange) compared with primary ovarian cancer ascites cells and primary ovarian cancer tumor samples, 43% are immune-related genes (highlighted in purple). A list of the differentially expressed genes (left) can also be found in Supplementary Table S3. **B** and **C,** GSEA comparing primary ascites (*n* = 8) and primary tumors (*n* = 14) shows enrichment in genes associated with IFNγ stimulation in primary ascites. **D–G,** Deconvolution analysis of the ovarian cancer RNA-seq dataset using Carcinoma Ecotyper to estimate the abundance of monocytes/macrophages (**D**), CD4^+^ T cells (**E**), CD8^+^ T cells (**F**), and cancer epithelial cells (**G**). Deconvolution can detect the absence of immune cells in the PDX samples. Note that certain predicted states (S) may not be specific to immune cells since they are abundant in the immunocompromised non-huPDX samples (e.g., monocyte/macrophage states S07–S09 and CD4^+^ T-cell states S03, S05, and S06). **G,** The relative abundance of epithelial states is also altered in the non-huPDX with a significant reduction in states S02–S04 based on an unpaired *t* test (*P* < 0.0001, *P* < 0.009, *P* < 0.005, respectively).

In a comparison of RNA-seq gene expression data from primary ovarian cancer and PDX samples, 43% of downregulated genes in PDX samples were either known macrophage markers (e.g.,CD68, CD163), involved in macrophage polarization (e.g., MALAT1, NEAT1, IL10RA) or responsible for immune infiltration (e.g., THBS1, THBS2, ITGA5, and collagens; Fig. 1A; Supplementary Table S3). Furthermore, RNA-seq analyses of unmatched ovarian cancer primary ovarian tumors and primary malignant ascites revealed an IFNγ-stimulated inflammatory macrophage signature shared across ascites samples but not in solid tumor samples (Fig. 1B and C). This supports the existence of two immune microenvironments, one in solid tumors and one in the ascites fluid. These distinct immune environments, which are absent in conventional PDX models, may influence ovarian cancer growth, dissemination, and response to therapies.

Deconvolution of bulk RNA-seq data can estimate the abundance of cell types and their states based on gene expression signatures obtained from single-cell RNA-seq databases. The Carcinoma Ecotyper program was used to analyze the cell type abundance by state in our ovarian cancer RNA-seq dataset including non-huPDX ascites and tumor samples and patient ascites and tumor samples (28). The estimated abundance by state of monocytes/macrophages, CD8^+^ T cells, CD4^+^ T cells, and epithelial cells is shown (Fig. 1D–G). As expected, non-huPDX ascites and tumor samples had a lower predicted abundance of monocyte/macrophage and T-cell populations over all. These samples also exhibited a skewing of epithelial cell states with a significant reduction in three epithelial cell states (states 2–4). Significant differences were also seen in the distribution of immune cell states in monocyte/macrophage and T-cell populations between patient ascites and patient tumors (Fig. 1D–G; Supplementary Fig. S1B–D) further supporting the hypothesis that there are distinct immune environments in ovarian cancer ascites versus solid tumors.

### Establishing Ovarian Cancer PDX Models with a Reconstituted Humanized Immune System

**FIGURE 2:**
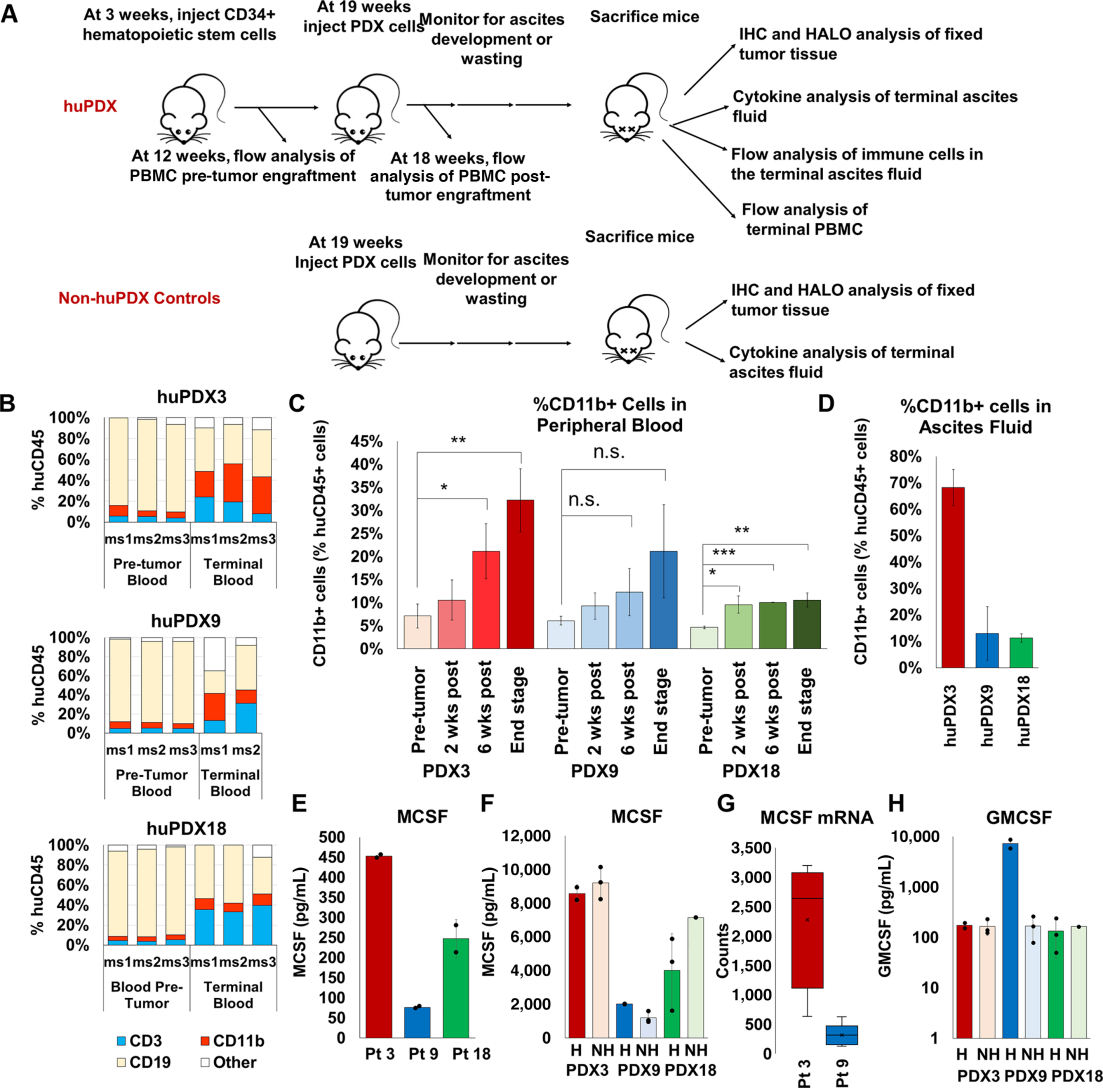
High M-CSF production influences the development of myeloid cells in huNBSGW PDX. **A,** Diagram of the huPDX study. **B,** Change in human CD45^+^ cell populations in peripheral blood before tumor engraftment versus end stage for each mouse (*n* = 3 mice/group). CD19^+^ B cells, CD3^+^ T cells, and CD11b^+^ myeloid cells make up the majority of human immune cells in the blood. Note that terminal samples from huPDX9 ms3 were not collected as the mouse died unexpectedly overnight. Mouse numbers are listed as ms1–3. **C,** Average percentage of human myeloid cells in the peripheral blood of huPDX before and after tumor challenge (values are the average ± SD). *P* values are based on an unpaired two-tailed Student *t* test. ***, *P* < 0.0005; **, *P* < 0.005; *, *P* < 0.05; n.s., not significant). **D,** Percentage of CD11b^+^ human myeloid cells in huPDX ascites fluid at end stage. **E,** M-CSF concentration in patient ascites fluid measured by ELISA. Samples were assayed in duplicate. Error bars are SD. **F,** M-CSF concentrations in PDX ascites fluid measured by cytokine array. **G,** M-CSF gene expression levels in patient samples as determined by RNA-seq analysis of primary patient samples and non-huPDX samples. **H,** GMCSF concentrations in PDX ascites fluid measured by cytokine array. Black dots represent the value for each sample. Bars are the average level and error bars are SEM. H = humanized NH = non-humanized.

To examine the influence of the tumor immune microenvironment on PDX disease progression, we chose three PDX models that demonstrated robust growth in immunocompromised mice, two platinum-sensitive PDXs (PDX3 and PDX18) and one platinum-resistant PDX (PDX9), to engraft in huNBSGW mice. Characteristics of the 3 patients are provided (Supplementary Table S2). The three PDXs were injected into the peritoneal cavity of huNBSGW mice engrafted with human cord blood–derived CD34^+^ HSCs from the same donor pool to establish a uniform immune background. All humanized mice exhibited a similar percent humanization of the peripheral blood before engraftment (Supplementary Fig. S2B). Non-huNBSGW injected with the same three PDXs served as non-huPDX controls. The huPDX study design is diagrammed in Fig. 2A.

### Engrafted Ovarian Cancer PDX Models Drive Increased Myeloid Cell Differentiation in huNBSGW Mice

Testing the impact of PDX tumor challenge on the development of engrafted human leukocytes revealed a striking change in the human CD45^+^ cell subpopulations in the peripheral blood of huNBSGW (Fig. 2B and C). Notably, tumor challenge led to a significant increase in the percentage of human myeloid cells as highlighted in Fig. 2C. Before tumor challenge, CD11b^+^ myeloid cells constituted on average 6% ± 1.8% of the human CD45^+^ cells in huNBSGW peripheral blood. At end stage, the average percentage of myeloid cells had risen to 21.3% ±10.8%. Myeloid cells made up a large proportion of human leukocytes within the ascites fluid of huPDX (Fig. 2D). huPDX3 had the highest percentage of human myeloid cells: 32% ± 6.8% of the human CD45^+^ cells in the peripheral blood and 68.2% ±11.3% in the ascites fluid. The increase in myeloid cells seen in huPDX3 was confirmed with another donor pool of CD34^+^ cells (Supplementary Fig. S2C) demonstrating that the increase in myeloid cells was not donor dependent, but instead driven by the tumor cells. CD3^+^ T cells increased over time with a concomitant decrease in human CD19^+^ B cells (Fig. 2B). Non–tumor-bearing huNBSGW showed similar changes over time (Supplementary Fig. S2D). Others have reported an increase in human T-cell percentage over time in CD34^+^ HSC-engrafted humanized models, indicating that the effect is not human tumor dependent, but a characteristic of CD34^+^ HSC-engrafted humanized models (35, 36). Thus, growth of ovarian cancer PDX tumors in huNBSGW does not affect T- and B-cell differentiation, but improves myeloid differentiation and results in the accumulation of myeloid cells in the peripheral blood and ascites fluid.

### Engrafted Ovarian Cancer PDX Models Have Elevated Levels of Human Cytokines in the Ascites Fluid

To characterize human cytokine profiles of huPDX models, end-stage acellular ascites fluid and blood plasma from huPDX and non-huPDX were analyzed on a 48-plex human cytokine/chemokine array. Plasma from a non–tumor-bearing huNBSGW mouse served as a control for the production of human cytokines by human immune cells in the absence of tumor cells. The control huNBSGW had high plasma levels (>100 pg/mL) of human IFNα2, IL12p40, MDC, MIG, and RANTES and lower but detectable levels of 10 other cytokines (Supplementary Fig. S3). Plasma from a non-humanized, non–tumor-bearing NBSGW served as a negative control to ensure that mouse cytokines were not detected by the human array. Thirty-five human cytokines had significantly higher levels in the PDX ascites fluid samples relative to plasma, demonstrating that cytokines produced in the ovarian cancer tumors are concentrated in the peritoneal microenvironment (Supplementary Table S4). Overall, cytokines were 2.42 log-concentration units higher in ascites versus plasma samples (S.E. = 0.30, *P* < 0.001). This agrees with a previous report finding higher concentration of cytokines in the ascites fluid versus plasma of patients with ovarian cancer (29).

### PDX Tumor Cells Produce Human M-CSF and GMCSF That can Influence Myeloid Differentiation

The observed increase in myeloid cells suggested that human cytokines produced by the ovarian cancer cells influence human immune cell differentiation within huPDX models. Humanized mice do not normally have robust myeloid cell differentiation due to the lack of cross-reactivity of mouse myeloid differentiation factors, mouse M-CSF and mouse GMCSF, with human receptors. Analyses of M-CSF cytokine levels in primary patient and PDX ascites detected high levels of human M-CSF in all samples (Fig. 2E and F). There were no significant differences in M-CSF levels comparing tumor-bearing huPDX and non-huPDX models (Fig. 2F), confirming that human M-CSF is produced by ovarian cancer cells. M-CSF secretion levels are correlated with transcription levels based on RNA-seq data analyses comparing the PDX models with the highest and lowest levels of human M-CSF, PDX3, and PDX9, respectively (Fig. 2G). GMCSF was also detected in the ascites fluid of all PDX mice (Fig. 2H). Finally, analysis of 40 patient ascites samples collected at surgery showed that nearly all samples had detectable M-CSF levels as measured by ELISA, and nearly half (19/40) had levels within the range of patients 3, 9, and 18 indicating that many ovarian cancer PDX cancer cells may express levels of M-CSF capable of stimulating myeloid differentiation (Supplementary Fig. S4). The data show that ovarian cancer huPDX models produce myeloid differentiation factors that improve human myeloid reconstitution beyond what has been characterized in non–tumor-bearing huNBSGW.

### huPDX Models Recapitulate the Cytokine Milieu of Ovarian Cancer Patient Ascites

**FIGURE 3:**
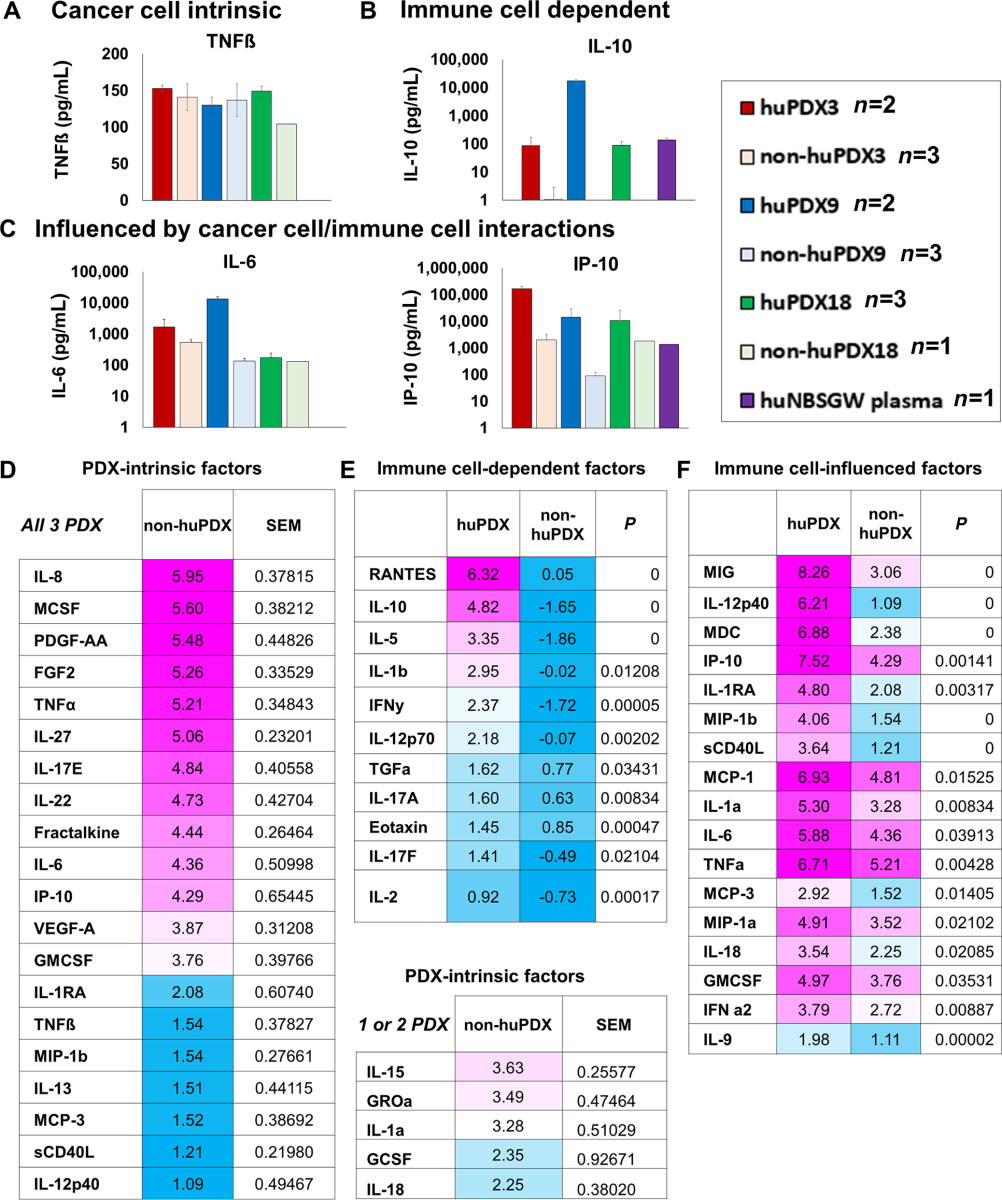
Analysis of human cytokine levels in huPDX and non-huPDX models indicates their origin. **A–C,** Examples of the subtypes of cytokines in huPDX. **A,** PDX-intrinsic cytokines. **B,** Immune cell–dependent cytokines. **C,** Immune cell–influenced cytokines. **D–F,** Tables of cytokine levels in ascites fluid. Levels are presented as the natural log of the least squares means (non-huPDX *n* = 7, huPDX *n* = 7) **D.** PDX-intrinsic cytokines (levels >1 in non-huPDX). Factors with detectable levels in only one or two PDX models are listed separately. **E,** Immune cell–dependent cytokines with non-huPDX levels <1. **F,** Factors with detectable levels in the non-huPDX, but significantly increased levels in the huPDX.

A large number of cytokines have been reported as elevated in ovarian cancer ascites samples including IL-6, IL-8, IL-10, IL-15, IP-10/CXCL10, MCP-1/CCL2, Mip1α/CCL3, Mip1β/CCL4, MDC, and VEGF (29, 30, 37). All of these cytokines were found at high levels in the huPDX ascites samples (Table 1), including chemokines that recruit immune cells to the tumor microenvironment such as MCP-1/CCL2 that recruits monocytes (38), MDC that recruits regulatory T cells (30), and MIG1 (CXCL9) and IP-10 (CXCL10) that recruit effector memory T cells (39, 40). Importantly, IL-10, an important immunosuppressive cytokine, is absent from the ascites fluid of non-huPDX models, but is detectable in all three huPDX models (Fig. 3B). Thus, the huPDX immune environment recapitulates that found in human patients with ovarian cancer.

**TABLE 1:**
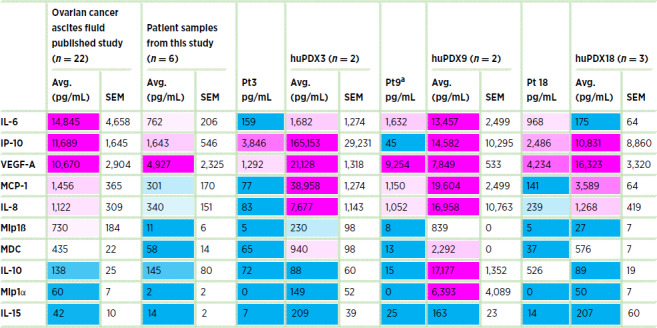
Cytokines elevated in the ascites fluid of patients with ovarian cancer are also present in the huPDX ascites fluid

NOTE: Levels of 48 cytokines and chemokines were measured in patient ascites fluid and huPDX ascites fluid by cytokine array. Values are expressed in pg/mL. Ovarian cancer ascites fluid from the published study are values from Giuntoli and colleagues (29) except MDC that is an estimate of the average from Wertel and colleagues (30). Ascites fluid samples from patients were collected at surgery. huPDX ascites samples were collected from mice at end stage. All cell-free ascites fluid samples were cryopreserved at −80°C prior to analysis. Color reflects the relative concentration levels from high (magenta) to low (blue). Note that the higher cytokine concentrations in huPDX samples may reflect increased levels at end stage disease.

^a^Pt 9 received neoadjuvant chemotherapy, which could alter cytokine levels in the patient ascites sample.

### Comparison of huPDX Versus non-huPDX Models Identifies Multiple Origins of Human Cytokine Production

In the huPDX models, human cytokines can be produced by the cancer cells or by the engrafted human immune cells. Comparison of huPDX and non-huPDX revealed three types of cytokine expression patterns: PDX-intrinsic cytokines, immune cell–dependent cytokines, and immune cell–influenced cytokines. Examples of all three types are shown in Fig. 3. PDX-intrinsic cytokines are produced by the cancer cells and were present in non-huPDX and huPDX ascites fluid (Fig. 3A). Twenty PDX-intrinsic cytokines were identified in all three PDX models (Fig. 3D). Other cytokines had significantly higher levels in the huPDX versus non-huPDX samples. Statistical analyses comparing the least squares means of cytokine levels in huPDX versus non-huPDX ascites samples demonstrated a significant increase in the levels of 28 cytokines in huPDX ascites. These cytokines were subdivided into immune cell–dependent factors that were only present in the huPDX samples (Fig. 3B) and immune cell–influenced factors that had lower levels in the non-huPDX with significantly higher levels in the huPDX (Fig. 3C). Cytokines detected in the PDX ascites fluid are listed in Fig. 3D and F. A large number of cytokines are PDX intrinsic, highlighting the potential for cancer cells to influence human immune cells within the tumor microenvironment. Human immune cells produce important cytokines such as IL-10 and increase the concentration of many cytokines within the ascites fluid emphasizing the impact of human immune cells on the tumor microenvironment.

### Human TAMs and Tumor-infiltrating Lymphocytes are Detected in huPDX Tumors

**FIGURE 4:**
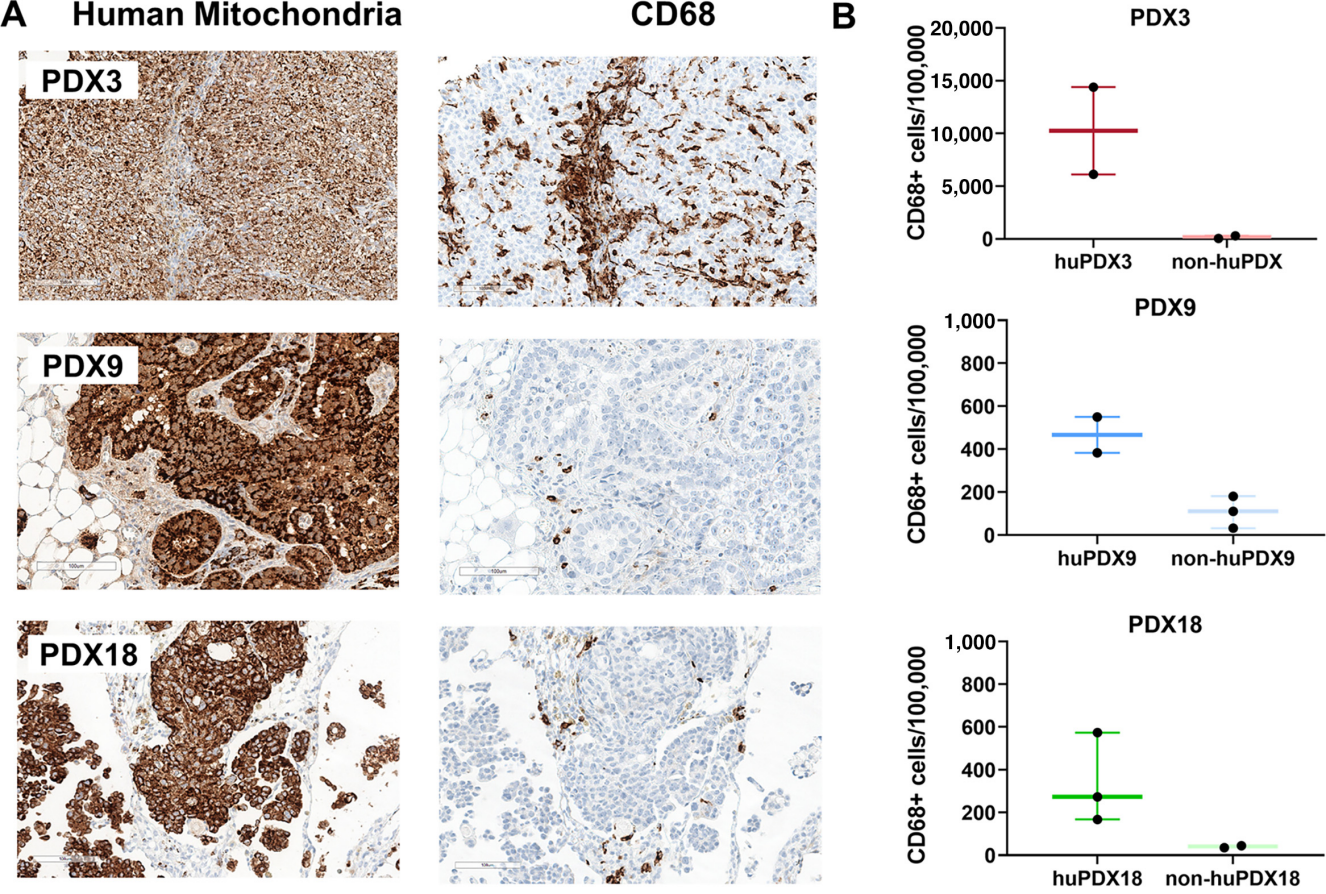
Detection of TAMs in huNBSGW PDX tumor sections. **A,** Serial sections of huNBSGW PDX tumor tissue were labeled with human-specific antibodies against a mitochondrial protein to label all human cells and the TAM marker CD68. Scale bar, 100 μm. **B,** Quantification of the number of TAMs using HALO software to identify CD68^+^ cells. Values are no. of positive cells/100,000 cells. The box plots indicate the mean, minimum, and maximum. Black dots indicate values for each tumor analyzed (*n* = 2–3).

**FIGURE 5:**
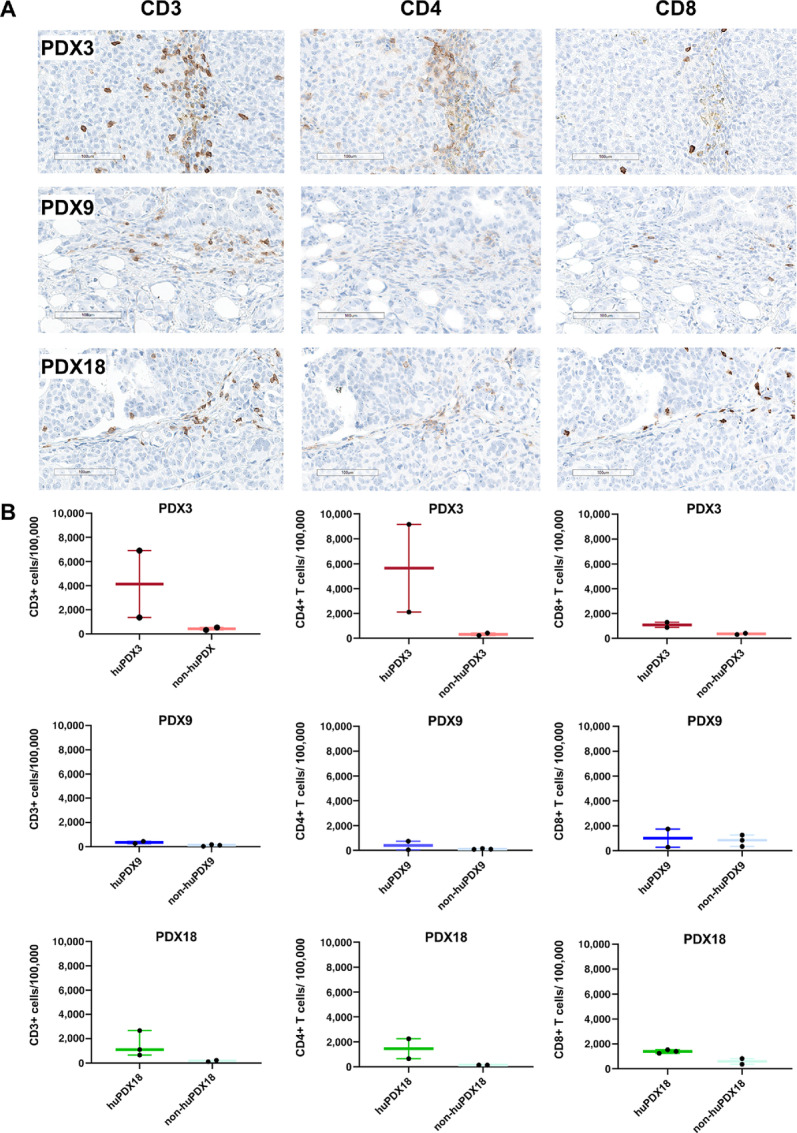
Detection of Human TILs in huNBSGW PDX tumor sections. **A,** Tumor sections from huPDX were labeled for human T-cell markers CD3, CD4, and CD8. Shown are scanned images of labeled sections at 20× magnification. Representative images are shown for huPDX3 and huPDX18. For huPDX9, the image shown is a rare group of TILs in a mostly negative section to show that the rare T cells were being labeled. Scale bar, 100 μm. **B,** Quantification of the number of TILs using HALO software. Values are reported as no. of positive cells/100,000 cells. The box plots indicate the mean, minimum, and maximum. Black dots indicate values for each tumor analyzed (*n* = 2–3).

Tumor-infiltrating immune cells can influence disease progression, patient prognosis, and response to therapies. To determine whether human immune cells were recruited to solid tumors, FFPE tumors from huPDX and non-huPDX mice were serially sectioned and labeled using human-specific immune cell markers to identify CD68^+^ TAMs (Fig. 4) and CD3^+^ tumor infiltrating lymphocytes (TILs) (Fig. 5). Quantification of labeled immune cells was performed using HALO software (Supplementary Fig. S5A). Non-huPDX tumor sections were used as negative controls (Supplementary Fig. S5B). Both CD68^+^ TAMs and CD3^+^ TILs were identified in tumors from all three PDX models. In huPDX3, TAMs had infiltrated into cancer cell clusters throughout the tumors, while in PDX9 and PDX18 TAMs were primarily localized to stromal tissue surrounding the cancer cell clusters (Fig. 4A). Quantification of the TAM populations showed that huPDX3 tumors had significantly more TAMs than huPDX18 using an unpaired *t* test (*P* < 0.0488; Fig. 4B). The total infiltrating CD3^+^ T-cell population as well as the CD4^+^ and CD8^+^ subpopulations were labeled in huPDX sequential tumor sections (Fig. 5A). Quantification of CD3^+^ TILs suggested that huPDX3 and huPDX18 may have more CD3^+^ TILs than huPDX9 (Fig. 5B), although the differences are not statistically significant due to variation in the individual tumor samples. Labeling sections for CD4^+^ and CD8^+^ T cells showed that huPDX3 TILs were primarily CD4^+^ (CD4^+^/CD8 average ratio of 4.7 ± 3.3), which correlates with poor survival in patients with ovarian cancer (41). huPDX18 had similar levels of CD4^+^ and CD8^+^ T cells (CD4^+^/CD8^+^ average ratio of 0.8 ± 0.6). huPDX9 TIL counts were minimal with counts comparable with the non-huPDX tumor controls. Though all three huPDX models had detectable TAMs and TILs, the density and localization of tumor-infiltrating immune cells appears to be PDX dependent.

**FIGURE 6:**
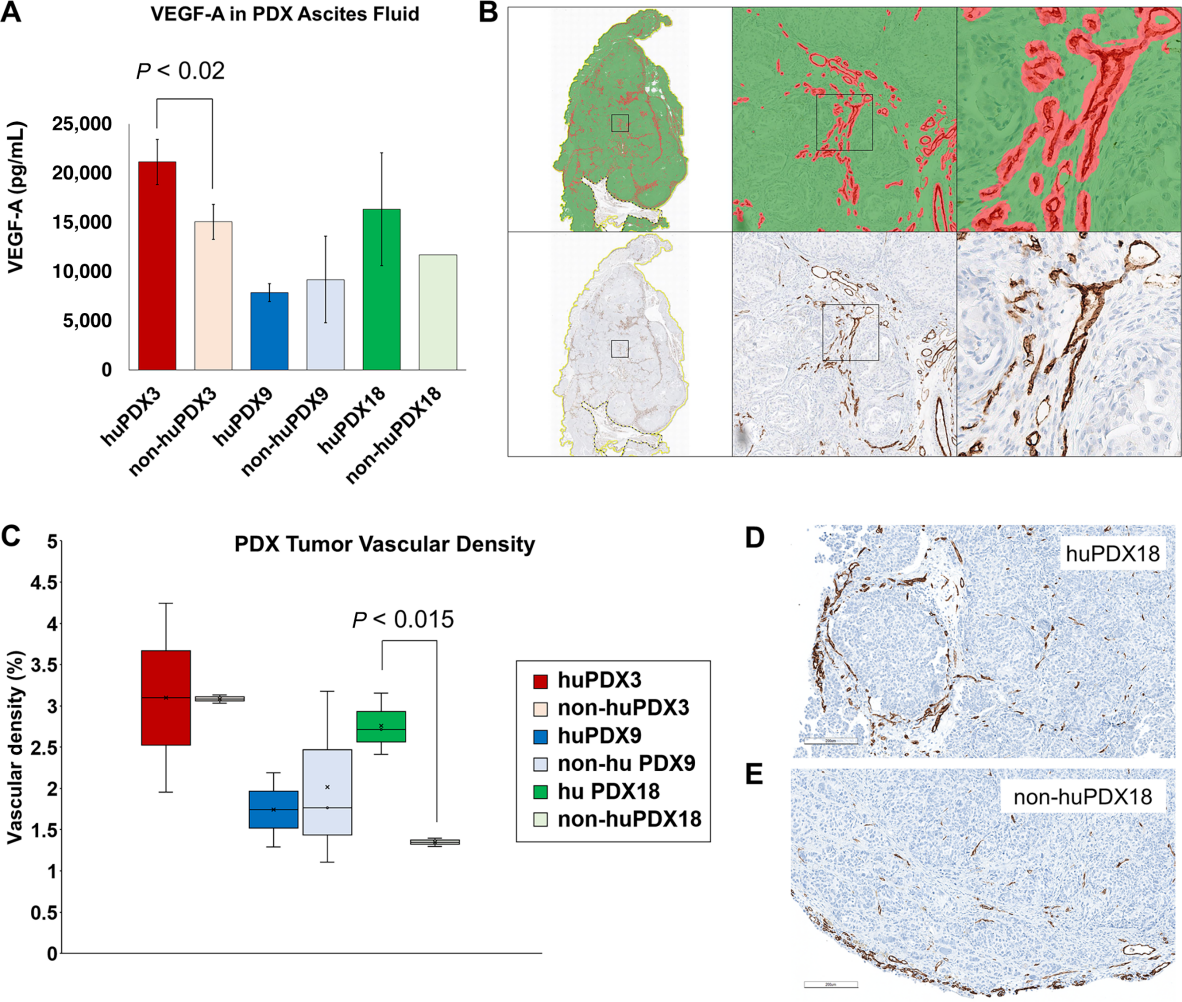
HALO analysis of tumor vascularization. **A,** The levels of VEGF-A in PDX ascites fluid was measured by ELISA. **B,** To quantify tumor vascularization in huPDX versus non-huPDX tumors, vessels were labeled with anti-CD31 antibody and vessel area was determined by analyzing scanned slides. The tumor tissue was masked on the basis of H&E staining (green) and CD31 labeled vessels were detected (red). Vessel density was calculated using the tissue classifier analysis software on the HALO platform. **C,** The quantification of vascular density (% vascular area) across whole sections of huPDX and non-huPDX samples. Values are the average vascular densities among samples (*n* = 2–3). huPDX18 sections showed increased vascular density compared to non-huPDX18 (*P* < 0.015, unpaired *t* test). **D** and **E,** Comparison of vascularity in representative images from huPDX18 (**D**) and non-huPDX18 (**E**) labeled with anti-CD31 antibody. Scale bar, 200 μm.

It was previously reported that the presence of TAMs can increase tumor vascularization by boosting VEGF-A production (42). Indeed, VEGF-A levels were significantly higher in the ascites of huPDX3 compared with non-huPDX3 and huPDX18 levels also trended higher (Fig. 6A). To examine whether tumor vascularization increased in the huPDX models, tumor sections were labeled with a CD31 antibody and vascular density was quantified using HALO analysis (Fig. 6B and C). PDX18 showed a significant increase in vascular density with humanization, suggesting that local VEGF production by human immune cells may have boosted VEGF levels enough to impact vascularization in this model (Fig. 6D and E). No change in vascular density was observed in PDX3 or PDX9. Therefore, further studies are needed to affirm a primary role for VEGF secretion by TAMs and tease out other factors that may be involved.

Analysis of huPDX tumors reveals that human immune cells can be recruited by PDX cells to recapitulate the tumor immune microenvironment in huPDX. Comparison of three huPDX models shows variability in immune cell recruitment and tumor vascularization that can be further explored in larger studies

### Engrafted Human Immune Cells Influence PDX Disease Progression

**FIGURE 7:**
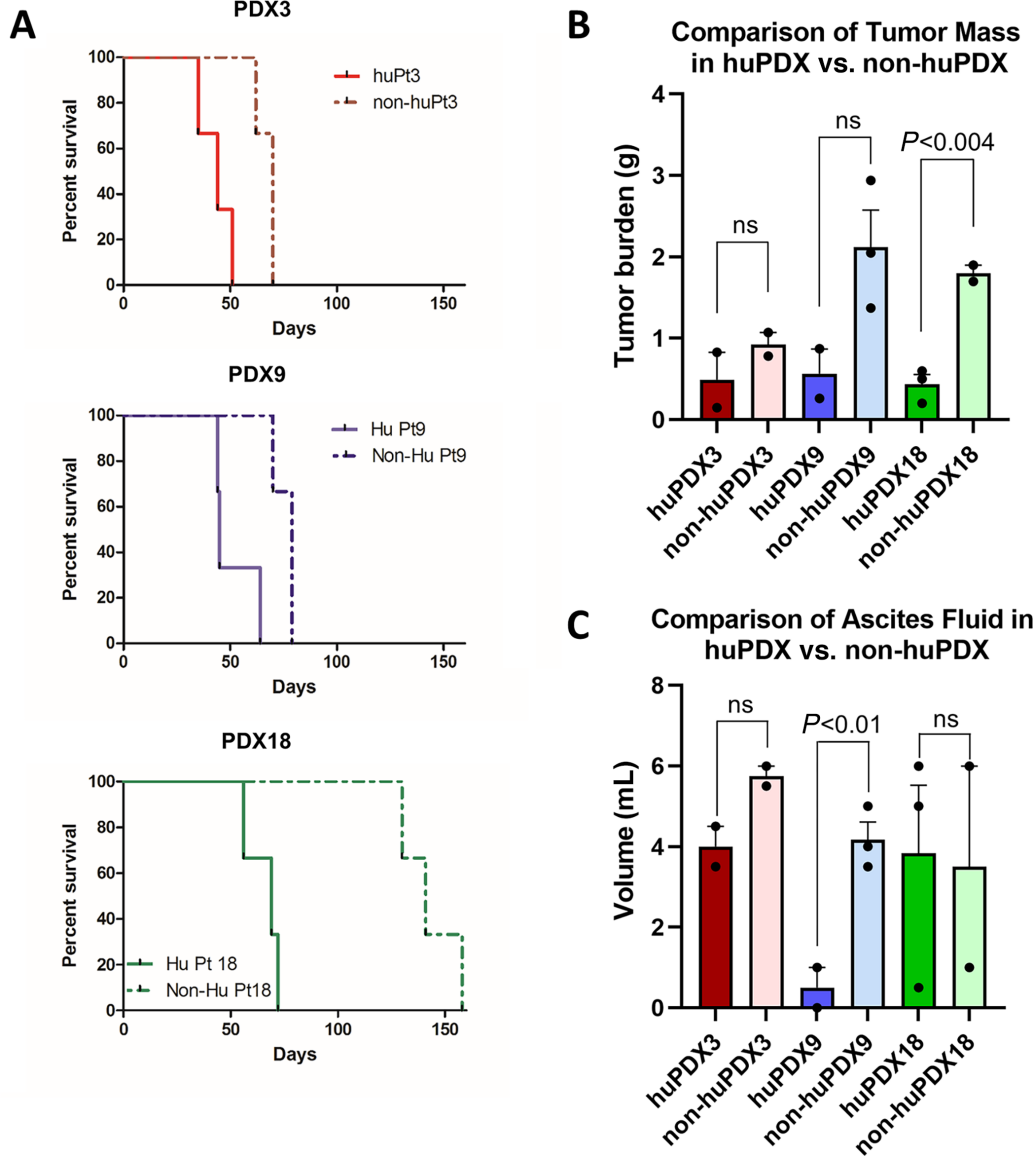
Survival time is reduced in huNBSGW PDX models. **A,** huNBSGW PDX3, 9, and 18 were engrafted with the same pooled donor CD34^+^ cells. Survival time was PDX dependent with PDX3 and 9 having a shorter survival time compared with PDX18. Survival curves are significantly different between huPDX and non-huPDX for all three PDX models (*P* < 0.025, log-rank test). For all PDX models, *n* = 3 mice/group for huPDX and non-huPDX. **B,** Solid tumor mass (in grams) of huPDX and non-huPDX at necropsy. Bars are the average tumor burden, error bars are SEM. Measures for individual mice are shown as black dots (*n* = 2–3 per group). **C,** Ascites fluid volume at necropsy. The ascites fluid volume trended higher in non-huPDX for PDX3 and PDX9, but was only significantly different for PDX9. Statistical analysis for **B** and **C** used unpaired *t* tests to compare huPDX versus non-huPDX values for each PDX line.

Similar to what is observed with the non-huPDX; survival times for the huPDX models were patient specific. PDX that consistently demonstrated shorter survival times in non-huNBSGW (PDX3 and PDX9), also showed more aggressive disease in huPDX. However, for all PDX models, huPDX mice had shorter survival times than the non-huPDX mice (Fig. 7A). NBSGW mice humanized with CD34^+^ HSCs have shown no evidence of GvHD in multiple previous studies (19, 43, 44) and our huPDX mice showed no clinical symptoms of GvHD (hair loss, dry skin, rapid weight loss). Therefore, the shorter survival times of the huPDX compared with the non-huPDX may indicate that the tumor-promoting role of human immune cells is dominant over the antitumor role in our ovarian cancer huPDX models. Tumors did not simply grow faster in huPDX mice, because the total tumor mass measured at endpoint was lower in huPDX compared with non-huPDX (Fig. 7B). Humanized PDX3 showed signs of anemia at end stage, likely due to enhanced myeloid differentiation (13), but all huPDX3 mice presented with malignant ascites prior to sacrifice indicating disease progression. Other disease characteristics with relevance to human disease contributed to early sacrifice in the huPDX mice. For instance, 2 of 3 huPDX9 mice presented with bowel obstruction prior to ascites development, while none of the non-huPDX9 mice showed evidence of bowel obstruction. All non-huPDX9 were sacrificed because of abdominal distension with ascites volumes similar to the other PDX groups (Fig. 7C). Because malignant bowel obstruction is a common complication of ovarian cancer affecting up to 51% of patients with recurrent disease, future studies with larger cohorts using huPDX9 will provide a model for studying the underlying causes of bowel obstruction in patients (45). huPDX18 accumulated malignant ascites much earlier than the non-huPDX18 requiring early sacrifice. Therefore, the influence of immune cells on ascites accumulation will be examined in future studies using the huPDX18 model. Reducing ascites accumulation is particularly important for ovarian cancer palliative care. The presence of human immune cells clearly influences disease progression in complex and PDX-specific ways. Collectively, the use of huPDX models affords new opportunities to examine disease relevant complications that may be driven by specific tumor cell characteristics.

## Discussion

In ovarian cancer, the importance of TAMs and myeloid-derived suppressor cells in disease progression, metastasis, and therapeutic response has been well established (8, 9, 46). Yet current patient-derived models for testing therapeutic response, cell line–derived xenografts and PDX in immunocompromised mice, lack a tumor immune microenvironment. Thus, preclinical testing can miss any immunomodulatory effects of novel therapies. The lack of an immune system in the non-huPDX models may also influence cancer cell gene expression patterns as is suggested by the deconvolution analysis of ovarian cancer gene expression data where significant differences were noted in the distribution of cancer epithelial cell states between the non-huPDX samples and the patient samples (Fig. 1G). The epithelial cell states that are significantly reduced in the non-huPDX models may be those that depend on immune cell cross-talk. To better reflect the immune microenvironment, recent studies have considered humanized mouse models. We detail the utility of a humanized mouse model of orthotopic ovarian cancer using the NBSGW mouse strain that obviates the need for pre-engraftment irradiation, while improving engraftment of human myeloid cells (19).

This is the first study reporting the use of huNBSGW mice for cancer research and the first to characterize a HIS mouse model of ovarian cancer. While this initial study tested a small number of mice per PDX, it uniquely examines human cytokine production by both non-huPDX and huPDX models across three PDX lines. Its provides the first proof of principle data that ovarian cancer PDX grown in humanized mice can model the immune microenvironment of patients with ovarian cancer including recapitulating the cytokine milieu of the ascites fluid and recruiting human TAMs and T cells to the PDX solid tumors, thereby filling an important unmet need among current animal models used to study ovarian cancer.

We demonstrate that ovarian cancer huPDX exhibit enhanced human myeloid cell differentiation due to the production of myeloid differentiation factors M-CSF and GMCSF by ovarian cancer cells. Therefore, our model reconstitutes myeloid cells without requiring constitutive expression of human cytokines by the mouse host. Apart from factors involved in differentiation and recruitment of macrophages, other human immune differentiation factors were detected in huPDX models, such as GCSF that is important for the activation of neutrophils and IL-15 that is responsible for the differentiation and activation of human NK cells. This suggests that our huPDX models may support a broader repertoire of human immune cells. Furthermore, we show PDX-specific recruitment of human CD68^+^ TAMs and CD3^+^ TILs to huPDX solid tumors. The ability of huPDX to significantly increase the human macrophage population and recruit TAMs and TILs to solid tumors provides opportunities for identifying immune responses to therapies in a model that more broadly represents the complexity of the human tumor microenvironment.

By comparing non-huPDX and huPDX ascites fluid, we were able to determine which human cytokines were produced intrinsically by the cancer cells, and which required human immune cells. In particular, we identified PDX-intrinsic production of human cytokines involved in the recruitment of macrophages to the tumor microenvironment. It is interesting to note that the PDX-intrinsic cytokines produced by ovarian cancer huPDX models are distinct from those produced by other cancer models producing high levels of IL-8, M-CSF, IP-10, MCP-1, VEGF-A, FGF-2, and PDGF-AA. A previous study using a 42-plex human cytokine array to analyze the serum of NSG mice engrafted with Nalm6-GFP leukemia cells reported high levels of PDX-intrinsic PDGF-AA and FLT-3 L as well as lower levels of FGF-2, VEGF-A, and TNFα (47). Tumors from humanized breast cancer models exhibited high levels of GMCSF, IL-6, IL-8, and TNFα (48). Thus, PDX-intrinsic cytokines may be specific to the tumor type.

Indeed, we find that cytokines previously identified in the ascites fluid of patients with ovarian cancer are present at elevated levels in patient ascites fluid and paired huPDX ascites fluid, showing that these models recapitulate the human ovarian cancer peritoneal environment. Many of these huPDX-expressed cytokines, such as MCP-1, IL-6, and IL-10, can support an immunosuppressive peritoneal environment. MCP-1 (CCL2) is the main cytokine responsible for recruitment of CCR2+ immunosuppressive macrophages to tumors (38, 49, 50). IL-6-dependent differentiation of CD4^+^ regulatory T cells has been stimulated by ovarian cancer ascites fluid *in vitro* (51). Meanwhile, IL-10 downregulates T-cell function and promotes immune tolerance (52). High IL-10 levels (>24 pg/mL in ascites fluid), detected in all three huPDX models, are associated with significantly shorter progression-free survival in patients with ovarian cancer (53, 54). These cytokines can also bind to receptors on the cancer cells and directly promote cancer invasion and metastasis (55–58).

The effect of humanization on disease progression, with huPDX reaching endpoint faster than the non-humanized controls, suggests that, in the absence of treatment, the protumor action of human immune cells dominates over antitumor immune responses. Previous work has noted that certain subcutaneous xenograft models grow faster in humanized mice, while others grow slower, indicating a heterogeneous immune response (7, 59). Our finding that multiple ovarian cancer PDXs have shorter survival times in humanized mice suggests that immune effects on disease progression may be tumor type dependent. Because the huPDX models demonstrate an increase in myeloid cells with tumor engraftment, future studies will examine the effect of myeloid populations on disease progression. PDX-intrinsic cytokine production may explain the observed influence of the immune system on disease progression.

Comparison of three huPDX models demonstrated that the degree of immune cell recruitment is a characteristic of the individual PDX model. PDX3 recruited larger numbers of TAMs, compared with PDX9 and PDX18. The localization of TAMs also varied among PDX models with TAMs in huPDX3 infiltrating into the epithelial tumor tissue, while TAMs in PDX9 and 18 remained within the stroma on the periphery of the tumors. PDX3 and PDX18 had higher numbers of T cells, while PDX9 had almost none. The high numbers of TILs in huPDX3 may be due to the high level of IP-10 (CXCL10). IP-10 has been reported to recruit TILs to ovarian cancer tumors (39). Future studies will examine whether differences in immune cell recruitment are dependent on patient genetic heterogeneity and differences in cancer cell expression of human cytokines and chemokines. Patient-specific differences in immune cell recruitment could be one of the factors influencing the variability in patient response to immunotherapies. This highlights the need to include multiple PDXs in preclinical studies to model the differences in patient immune response.

Collectively, our data show that huPDX models in NBSGW mice reconstitute human myeloid cells and T cells within the tumor microenvironment providing more clinically relevant models to examine cancer cell/immune cell interactions and test novel therapeutics. Our huPDX models are ideal for testing the response of genetically diverse ovarian cancer PDX to anticancer therapies including immunomodulatory agents such as immune checkpoint inhibitors and TAM targeting therapies.

## Supplementary Material

Table S1 Antibodies used for flow cytometry and IHC analysis

Table S2 Patient characteristics for the three PDX models

Table S3 Genes with lower expression in non-humanized PDX ovarian cancer samples compared to patient ovarian cancer samples

Figure S1 Gene expression analysis of OvCa samples

Figure S2 Analysis of humanization in mice engrafted with CD34+ hematopoietic stem cells

Figure S3 Levels of human factors in the plasma of a non-tumor bearing humanized NBSGW and a non-humanized non-tumor bearing NBSGW control.

Figure S4 Levels of human M-CSF detected in patient ascites fluid

Figure S5 HALO Quantification of immune cells

Table S4 Human cytokines with significantly higher levels in PDX ascites samples compared to plasma samples
