# Supplementary material for: Humanized Patient-derived Xenograft Models of Disseminated Ovarian Cancer Recapitulate Key Aspects of the Tumor Immune Environment within the Peritoneal Cavity
Source: Cancer Res Commun. 2023 Feb 22;3(2):309–24. doi: 10.1158/2767-9764.CRC-22-0300 (PMC9973420; doi:10.1158/2767-9764.CRC-22-0300)
Supplement: Table S1 — Antibodies used for flow cytometry and IHC analysis [file crc-22-0300-s01.pdf]

Supplementary Data

Supplementary Table S1. Antibodies used for flow cytometry and IHC analysis.

Antibody specificity	Cell type	Fluorescence	Antibody Clone	Supplier	RRID	Use
Mouse CD45	Mouse PBMCs	FITC	30-F11	BD Bioscience	AB_394610	Flow cytometry
Human CD45	Human PBMCs	APC	HI30	BD Bioscience	AB_2916374	Flow cytometry
Human CD3	Human T cells	BV421	UCHT1	BD Bioscience	AB_2744383	Flow cytometry
CD11b	Human myeloid cells	PECy7	ICRF44	BD Bioscience	AB_396849	Flow cytometry
Human CD19	Human B cells	PE	HIB19	BD Bioscience	AB_395813	Flow cytometry
Human mitochondria	All human cells		MAB1273	Sigma	NA	IHC
Human CD68	Human TAMs		PG-M1	Agilent Dako	NA	IHC
Human CD3	Human T cells		RBT-CD3	Bio SB	NA	IHC
Human CD4	Human T cells		SP35	Sigma	NA	IHC
Human CD8	Human T cells		SP57	Ventana	AB_2335985	IHC
Mouse CD31	Mouse endothelial cells		polyclonal	Abcam	ab124432	IHC
