# Supplementary material for: Humanized Patient-derived Xenograft Models of Disseminated Ovarian Cancer Recapitulate Key Aspects of the Tumor Immune Environment within the Peritoneal Cavity
Source: Cancer Res Commun. 2023 Feb 22;3(2):309–24. doi: 10.1158/2767-9764.CRC-22-0300 (PMC9973420; doi:10.1158/2767-9764.CRC-22-0300)
Supplement: Table S2 — Patient characteristics for the three PDX models [file crc-22-0300-s02.pdf]

Supplementary Table S2. Patient characteristics for the three PDX models

Patient ID#	Diagnosis	Age at diagnosis	Platinum sensitivity	BRCA mutation status	Survival time after surgery
**PDX3**	HGSOC Stage 3c	49	Sensitive	wildtype	survived 6.5 years, DOD
**PDX9**	HGSOC Stage 3B	56	Resistant	wildtype	survived 18 months, DOD
**PDX18**	HGSOC Stage 3C	60	Sensitive	wildtype	recurrence at 1 year, lost to follow up

** DOD: dead of disease*
