# Supplementary material for: Humanized Patient-derived Xenograft Models of Disseminated Ovarian Cancer Recapitulate Key Aspects of the Tumor Immune Environment within the Peritoneal Cavity
Source: Cancer Res Commun. 2023 Feb 22;3(2):309–24. doi: 10.1158/2767-9764.CRC-22-0300 (PMC9973420; doi:10.1158/2767-9764.CRC-22-0300)
Supplement: Table S3 — Genes with lower expression in non-humanized PDX ovarian cancer samples compared to patient ovarian cancer samples [file crc-22-0300-s03.pdf]

Supplementary Table S3. Genes with lower expression in non-humanized PDX ovarian cancer samples compared to patient ovarian cancer samples

Gene Symbol	Immune related	Role
THBS1	Yes	Macrophage polarization/activation and immune infiltration
COL1A1	Yes	Immune infiltration
COL3A1	Yes	Immune infiltration
COL1A2	Yes	Immune infiltration
MALAT1	Yes	Macrophage polarization/activation
NEAT1	Yes	Macrophage polarization/activation
ITGA5	Yes	Immune infiltration
CDKN1A	Yes	Macrophage polarization/activation
LOXL2	No	
ALDH1A3	No	
PTGIS	Yes	Macrophage polarization/activation
LOXL2	No	
DAB2	No	
SERPINE1	Yes	NK cell marker
LAMA4	No	
ADAMTS2	Yes	Expressed in active macrophages
THBS2	Yes	Immune infiltration
COL5A2	Yes	Immune infiltration
ACTA2	No	
TGFBI	No	
CCDC80	No	
ITGAX	Yes	Macrophage/dendritic cell marker
CSF1R	Yes	Macrophage marker
CD14	Yes	Macrophage marker
CYP27A1	No	
IL10RA	Yes	Macrophage marker
LSP1	No	
CD68	Yes	Macrophage marker
SPP1	No	
DPP4	No	
LRRN4	No	
CA12	No	
HLA-DRB5	Yes	Macrophage marker
LYZ	No	
MIAT	No	
PDGFRA	No	
SVEP1	No	
ABI38P	No	
PLAC4	No	
SYNPO2	No	
HMCN1	No	
MMP11	No	
C1QC	Yes	Serum complement system
C1QA	Yes	Serum complement system
VSIG4	No	
C1QB	Yes	Serum complement system
CD163	Yes	Macrophage marker
LAPTM5	No	
CYBB	Yes	Phagocytosis
STAB1	No	
PTPRC	No	
EMILIN1	No	
SLCO2B1	No	
ZEB2	No	
VCAM1	Yes	Cytokine-activated endothelium
LUM	No	
DCN	No	
MEG3	No	
COL6A3	Yes	Immune infiltration
CDH11	No	
POSTN	No	
MARCO	Yes	Macrophage marker
SERPINB2	Yes	Expressed in active macrophages

Purple indicates immune related genes.
