# Supplementary material for: Humanized Patient-derived Xenograft Models of Disseminated Ovarian Cancer Recapitulate Key Aspects of the Tumor Immune Environment within the Peritoneal Cavity
Source: Cancer Res Commun. 2023 Feb 22;3(2):309–24. doi: 10.1158/2767-9764.CRC-22-0300 (PMC9973420; doi:10.1158/2767-9764.CRC-22-0300)
Supplement: Figure S1 — Gene expression analysis of OvCa samples [file crc-22-0300-s04.pdf]

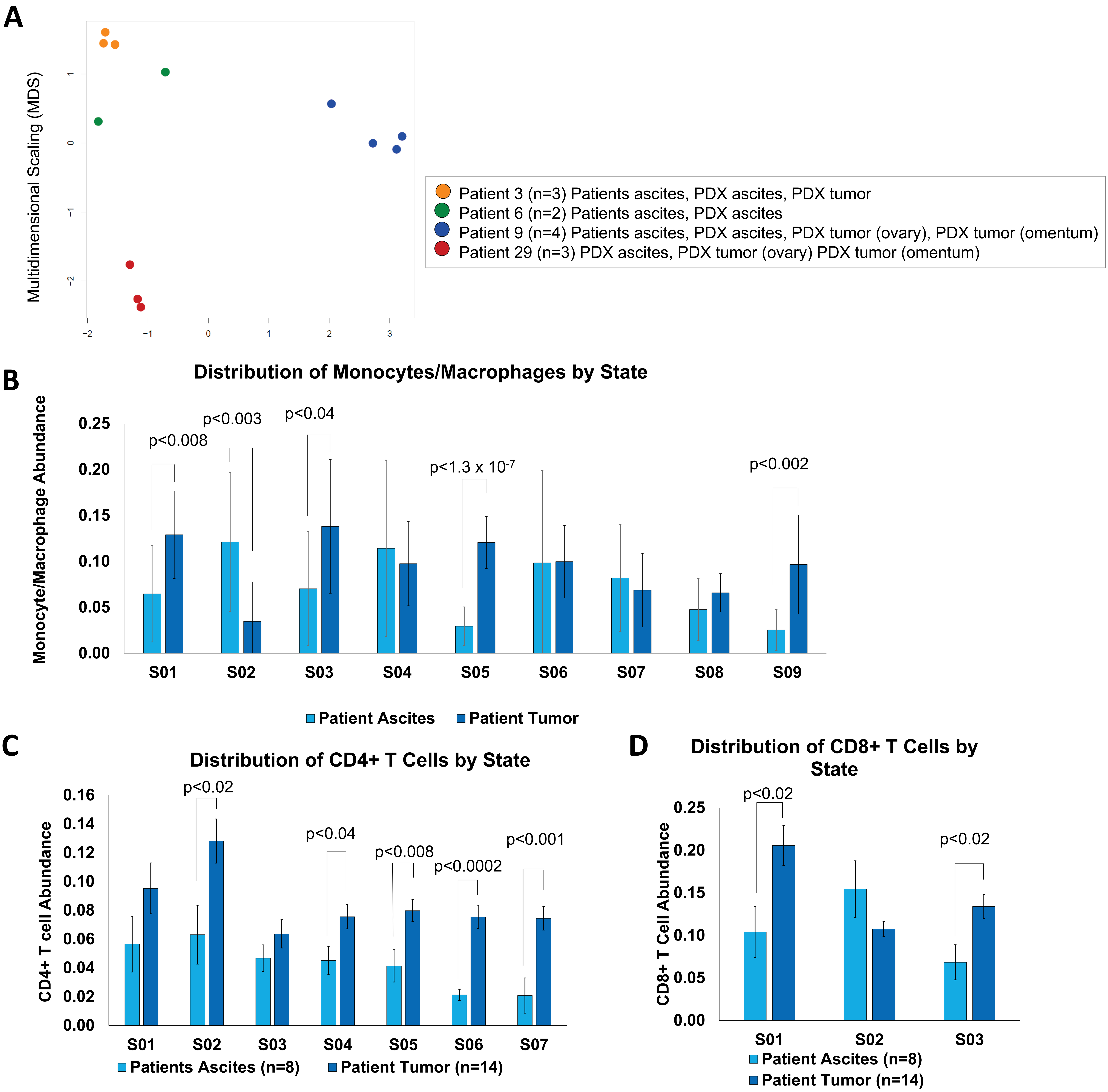

Fig. S1 Gene expression analysis of OvCa samples. A. Multidimensional Scaling (MDS) plot of expression data comparing patient samples and their matching PDX samples. Patient samples and PDX samples originating from the same patient cluster together. **B-D.** Deconvolution analysis of OvCa normalized gene expression data. Normalized gene expression data was analyzed using the Carcinoma Ecotyper software (https://ecotyper.stanford.edu/carcinoma) that assigns cell abundance and cell states to expression data. Distribution of states is compared between patient ascites samples (n=8) and patient tumor samples (n=14). States are determined based on gene expression clusters pre-specified by the program. The bars represent the average abundance for each state (S01-S09) +/- SEM. P-values denote significant differences in state abundance based on unpaired t-tests. **A-C.** The average estimated abundance of monocyte/macrophage (**A**), CD4+ T cell (**B**) and CD8+ T cell (**C**) states in patient ascites versus patient tumor samples.
