# Supplementary material for: Humanized Patient-derived Xenograft Models of Disseminated Ovarian Cancer Recapitulate Key Aspects of the Tumor Immune Environment within the Peritoneal Cavity
Source: Cancer Res Commun. 2023 Feb 22;3(2):309–24. doi: 10.1158/2767-9764.CRC-22-0300 (PMC9973420; doi:10.1158/2767-9764.CRC-22-0300)
Supplement: Figure S2 — Analysis of humanization in mice engrafted with CD34+ hematopoietic stem cells [file crc-22-0300-s05.pdf]

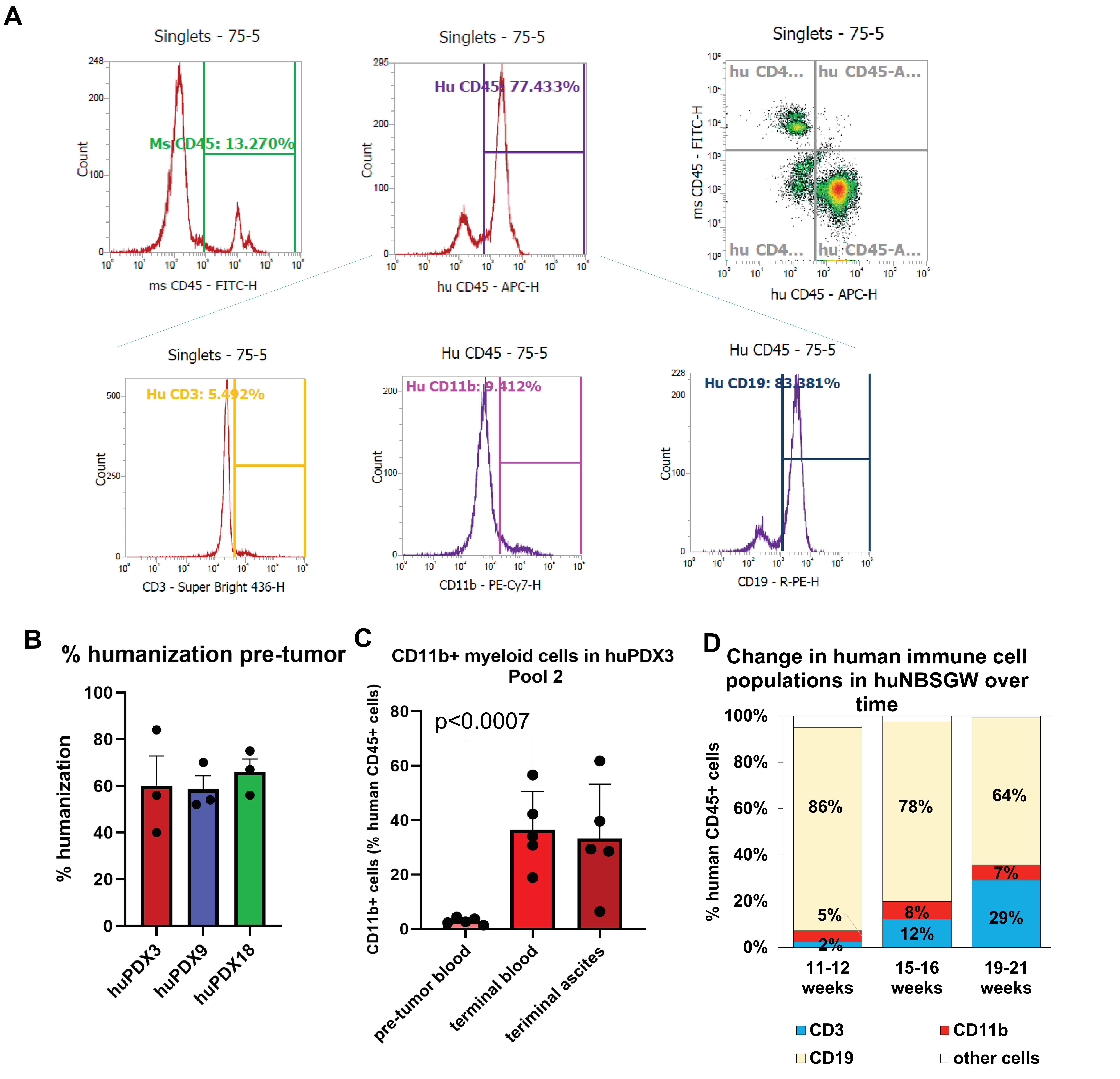

Fig. S2 Analysis of humanization in mice engrafted with CD34+ hematopoietic stem cells. Multi-color flow cytometry identified mouse and human CD45+ immune cells. Gating on human CD45+ blood cells, the human cells are then analyzed to determine the T cell (CD3+), B cell (CD19+) and myeloid cell (CD11b+) populations. **B.** Similar humanization in the peripheral blood from huNBSGW mice pre-PDX engraftment. The % humanization is the percent of human CD45+ cells in all CD45+ cells (mouse and human). There is no significant difference in humanization among the groups ($p<0.8$ by one-way ANOVA). **C**. Change in the human immune profile of huNBSGW mice over time is marked by an increase in % human T-cells (CD3+) and a decrease in % human B-cells (CD19+). Myeloid cells (CD11b+) remain stable at 5-8%. The analysis is based on flow cytometry labeling of peripheral blood samples collected from huNBSGW at specified times post-engraftment. **D**. Consistent increase in myeloid cells with tumor engraftment in huNBSGW PDX3. PDX3 (M-CSF high) demonstrates an increase in myeloid cells in peripheral blood at end point with a second independent donor pool of CD34+ cells.
