# Supplementary material for: Humanized Patient-derived Xenograft Models of Disseminated Ovarian Cancer Recapitulate Key Aspects of the Tumor Immune Environment within the Peritoneal Cavity
Source: Cancer Res Commun. 2023 Feb 22;3(2):309–24. doi: 10.1158/2767-9764.CRC-22-0300 (PMC9973420; doi:10.1158/2767-9764.CRC-22-0300)
Supplement: Figure S3 — Levels of human factors in the plasma of a non-tumor bearing humanized NBSGW and a non-humanized non-tumor bearing NBSGW control. [file crc-22-0300-s06.pdf]

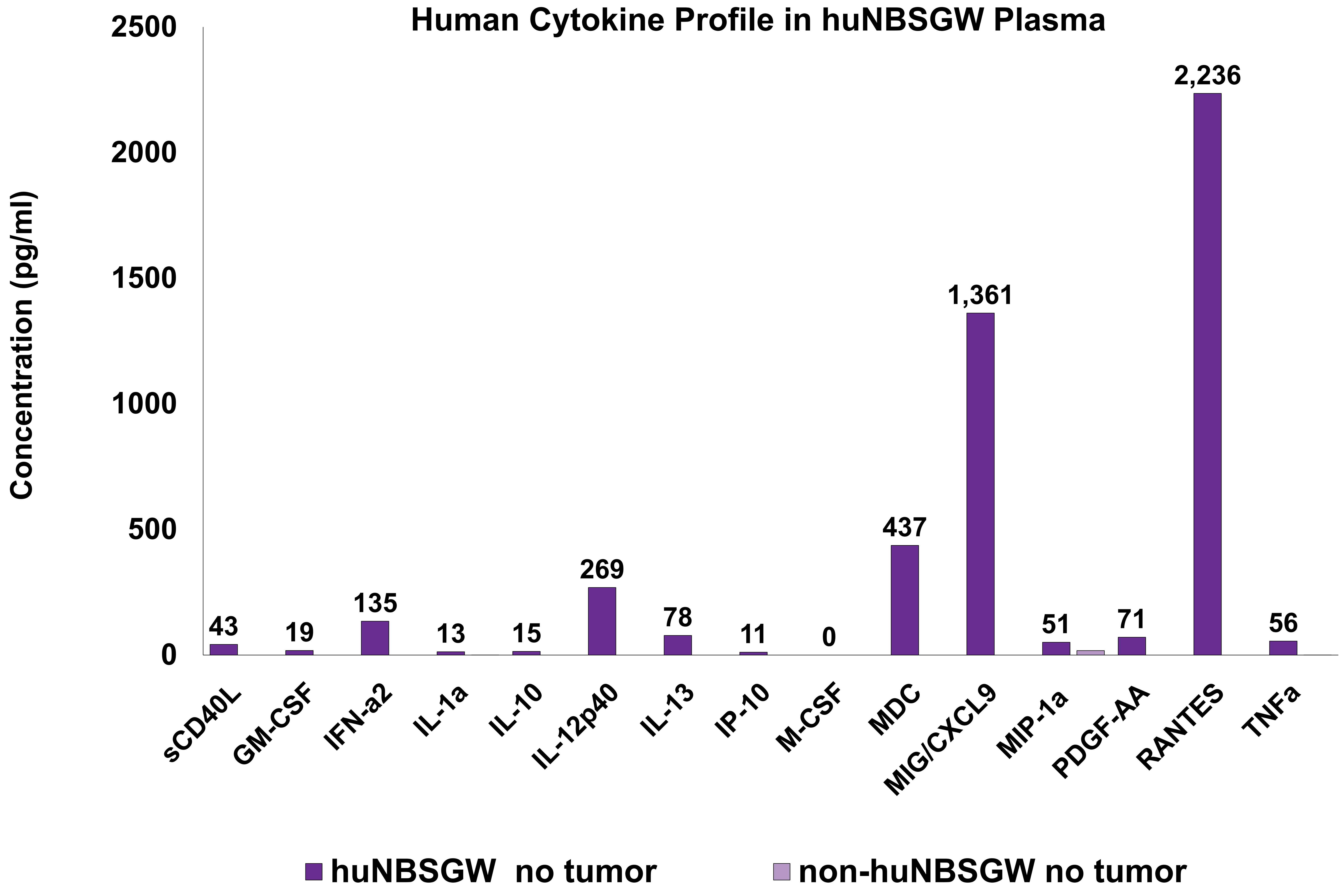

Fig. S3 Levels of human factors in the plasma of a non-tumor bearing humanized NBSGW and a non-humanized non-tumor bearing NBSGW control. Levels were measured on a 48-plex human cytokine/chemokine array.
