# Supplementary material for: Humanized Patient-derived Xenograft Models of Disseminated Ovarian Cancer Recapitulate Key Aspects of the Tumor Immune Environment within the Peritoneal Cavity
Source: Cancer Res Commun. 2023 Feb 22;3(2):309–24. doi: 10.1158/2767-9764.CRC-22-0300 (PMC9973420; doi:10.1158/2767-9764.CRC-22-0300)
Supplement: Figure S4 — Levels of human M-CSF detected in patient ascites fluid [file crc-22-0300-s07.pdf]

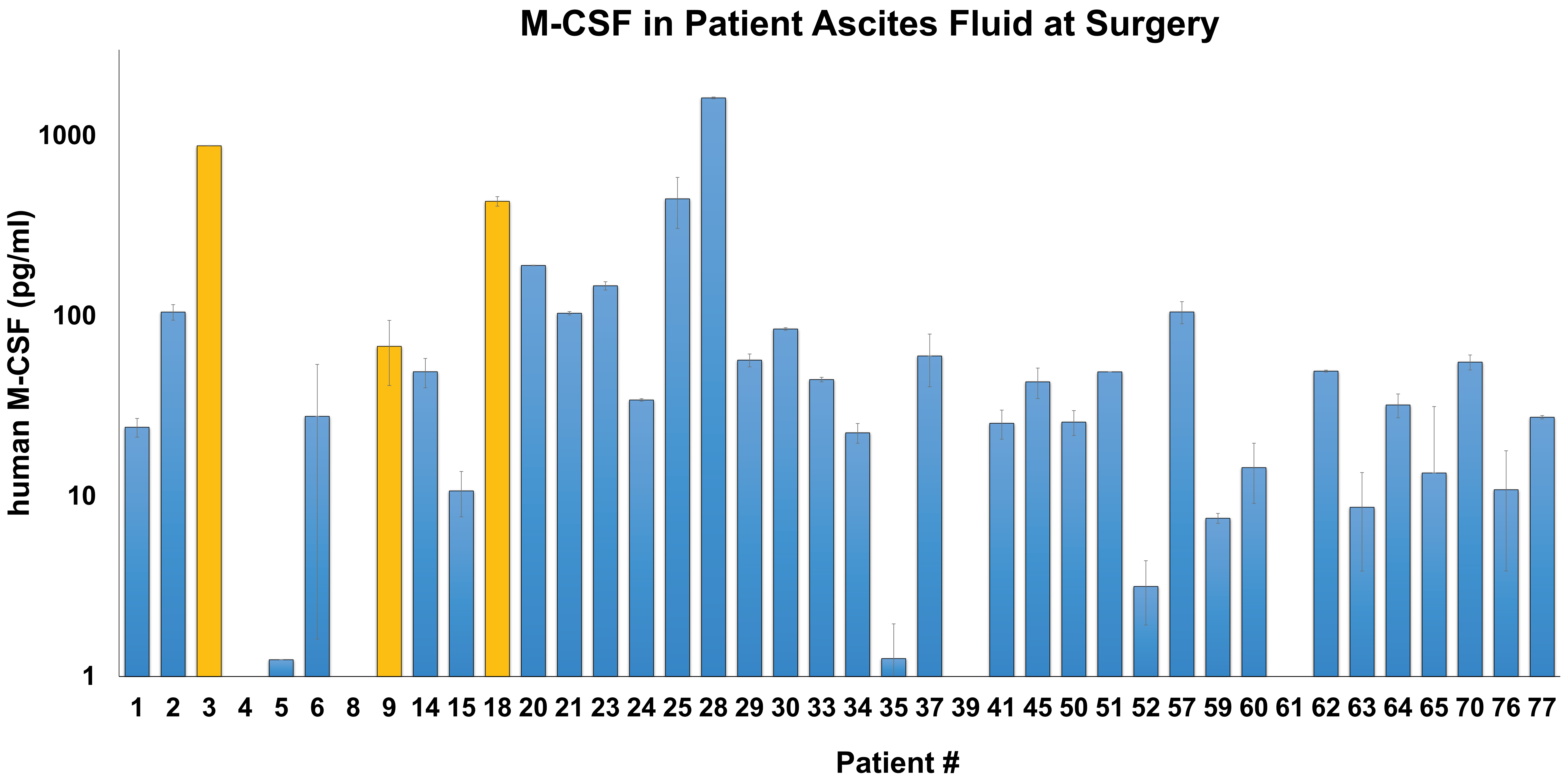

Fig. S4 Levels of human M-CSF detected in patient ascites fluid. Cell-free ascites fluid collected at surgery from 40 ovarian cancer patients was tested for human M-CSF levels by ELISA. Bars are the average of two replicates (+/- STDEV). M-CSF was detectable in 36 of 40 patient samples. Patient samples from which the three PDX used in this study originated are shown in yellow. M-CSF is detectable in 36 of the 40 samples and falls within or above the range of patients 3,9, and 18 in 19 samples.
