# Supplementary material for: Humanized Patient-derived Xenograft Models of Disseminated Ovarian Cancer Recapitulate Key Aspects of the Tumor Immune Environment within the Peritoneal Cavity
Source: Cancer Res Commun. 2023 Feb 22;3(2):309–24. doi: 10.1158/2767-9764.CRC-22-0300 (PMC9973420; doi:10.1158/2767-9764.CRC-22-0300)
Supplement: Figure S5 — HALO Quantification of immune cells [file crc-22-0300-s08.pdf]

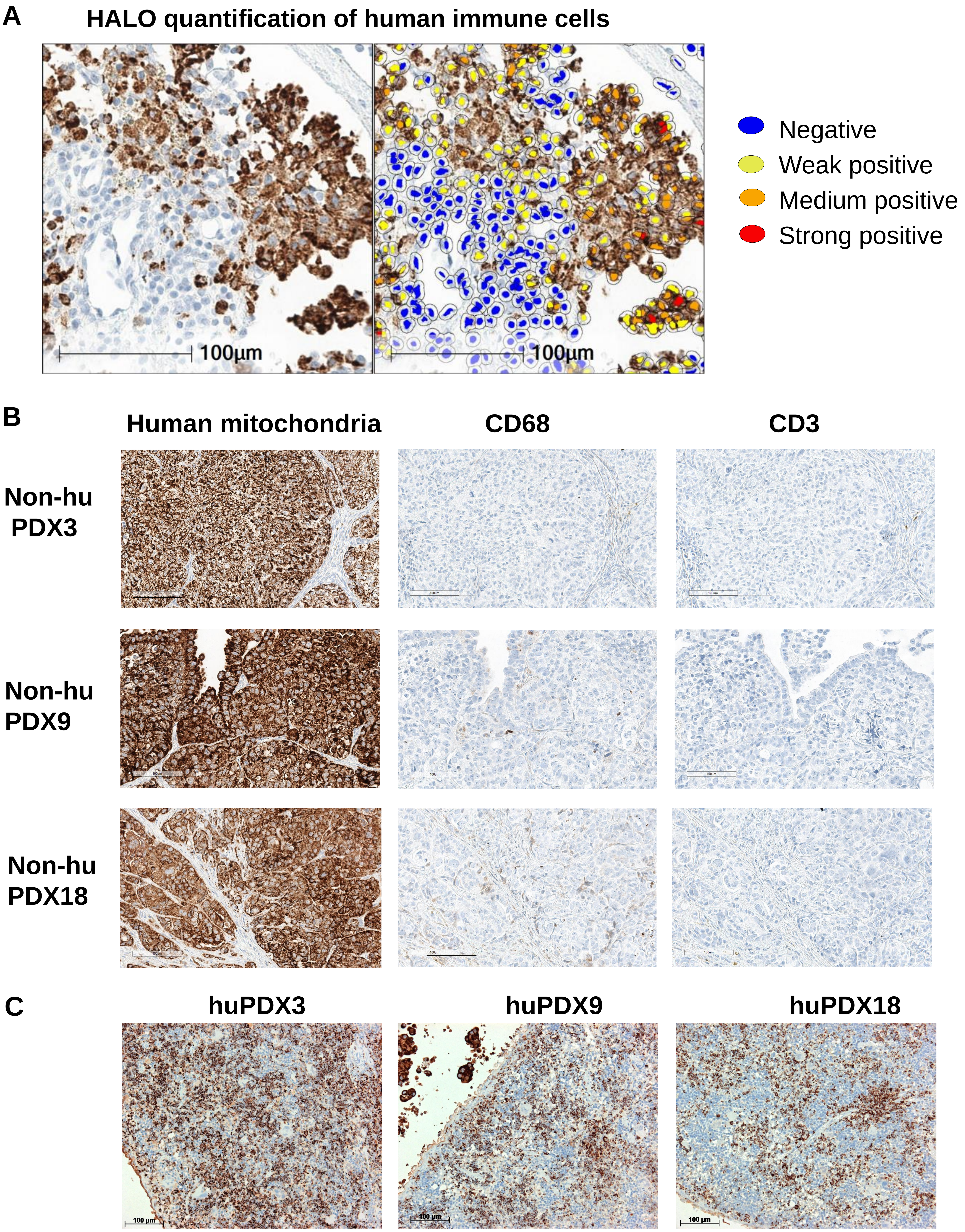

Fig. S5. HALO Quantification of immune cells and vascular density. A. An example of HALO quantification of human immune cells in a huPDX tumor. All cells within a tumor section are detected and then each cell is marked as negative or positive (weak, medium, strong) based on the analysis parameters. Scale bar=100 µm **B.** Immunohistochemistry of non-huPDX control tumors. The human mitochondrial marker labels all PDX cancer cells. The human-specific markers for CD68 and CD3 show little to no background staining. **C.** Spleens from huPDX have populations of human immune cells labeled with the human mitochondria marker. Dark staining of cancer spheroids is also visible in the huPDX9 section (upper left). Scale bars=100 µm.
