# Supplementary material for: Humanized Patient-derived Xenograft Models of Disseminated Ovarian Cancer Recapitulate Key Aspects of the Tumor Immune Environment within the Peritoneal Cavity
Source: Cancer Res Commun. 2023 Feb 22;3(2):309–24. doi: 10.1158/2767-9764.CRC-22-0300 (PMC9973420; doi:10.1158/2767-9764.CRC-22-0300)
Supplement: Table S4 — Human cytokines with significantly higher levels in PDX ascites samples compared to plasma samples [file crc-22-0300-s09.pdf]

Supplementary Table S4. Human cytokines with significantly higher levels in PDX ascites samples compared to plasma samples. Factors are ordered based on ascites levels high to low. Levels are the log squares mean of all PDX ascites or plasma samples. Color scale shows relative levels with pink high and blue low.

Cytokine	Ascites LSMeans	SEM	Plasma LSMeans	SEM	p-value
M-CSF	8.2376	0.3515	2.9882	0.38212	0
IP-10	7.975	0.60201	3.8294	0.65445	0.00011
IL 8	7.6304	0.34784	5.0821	0.37815	0.00005
MCP1	6.8919	0.54794	4.8412	0.59567	0.01856
FGF_2	6.8143	0.30842	4.2355	0.33529	0.00001
TNFα	6.6726	0.32051	5.2466	0.34843	0.00621
PDGF AA	6.5517	0.41234	3.8245	0.44826	0.00017
IL 27	6.3871	0.21342	4.2122	0.23201	0
IL 1RA	6.3749	0.55872	0.5132	0.6074	0
MIG	6.2657	0.21417	5.0478	0.23283	0.00082
IL 6	6.2248	0.46911	4.0099	0.50998	0.00401
IL 1a	6.1278	0.4694	2.4501	0.51029	0.00002
GM_CSF	5.546	0.36579	3.1804	0.39766	0.00022
Fractalkine	5.5079	0.24343	2.884	0.26464	0
IL 17E	5.3828	0.37308	3.7895	0.40558	0.00823
GROa	5.3238	0.47464	2.9213	0.4366	0.00111
IL 15	4.9463	0.23527	2.6377	0.25577	0
TNFß	4.9295	0.28047	-1.1915	0.43575	0
IL13	4.9114	0.4058	-0.7923	0.44115	0
IL 18	4.6658	0.34973	1.1234	0.3802	0
IFNα2	4.266	0.25303	2.2373	0.27508	0.00002
TGFα	3.381	0.25799	-0.9906	0.28047	0
MIP 1ß	3.3587	0.25445	2.2372	0.27661	0.00663
MCP3	3.1507	0.35591	1.2868	0.38692	0.00173
FLT3L	2.8899	0.41323	-1.1633	0.44923	0
IL 1b	2.8442	0.73826	0.0917	0.80258	0.01896
sCD40L	2.8064	0.20218	2.0481	0.2198	0.01834
IL 4	2.6451	0.03536	-1.2447	0.03844	0
EGF	1.7943	0.26219	-0.8321	0.28503	0
IL 17F	1.7066	0.51958	-0.7846	0.56484	0.00356
Eotaxin	1.6082	0.10024	0.6938	0.10897	0
IL 7	1.2489	0.23602	0.2319	0.25658	0.00775
IL 5	1.2036	0.29227	0.2869	0.31773	0.04469
IL 2	0.7929	0.24787	-0.6055	0.26946	0.00088
